# Resisting *Xylella fastidiosa*: xylem anatomical changes in the susceptible olive cultivar Cellina di Nardò after long‐term infection

**DOI:** 10.1111/plb.70210

**Published:** 2026-03-25

**Authors:** E. Sabella, D. Greco, E. Nutricati, A. Frontini, L. De Bellis, M. Vergine, A. Luvisi

**Affiliations:** ^1^ Department of Biological and Environmental Sciences and Technologies University of Salento Lecce Italy; ^2^ National Biodiversity Future Center Palermo Italy

**Keywords:** fluorescence *in situ* hybridization, *Olea europaea*, vascular pathogens, xylem anatomy, xylem vessel arrangement

## Abstract

- The *Xylella fastidiosa* subsp. *pauca* (*Xfp*) disease has destroyed the olive cultivations of Salento, a peninsula in the extreme south‐east of Italy, and is dangerously moving northwards. Cellina di Nardò is among the cultivars most susceptible to the pathogen. However, in recent years, spontaneous canopy restoration was observed in some naturally infected plants.
- In this work, we investigated xylem morphological changes, resembling a response to drought stress, in plants of the susceptible cultivar Cellina di Nardò more than 10 years after the appearance of *Xfp* in Apulia.
- Results showed a significant reduction in xylem vessel diameter and frequency in 1‐year‐old branches, two anatomical traits already associated to *Xfp* resistance in several species, as well as a change in vessel placement and in the number of stand‐alone (or solitary) xylem vessels. In fact, confocal microscope studies demonstrated a rise in stand‐alone vessels while decreasing the number of clustered vessels.
- Reduced contact points in the xylem network can slow down the pathogen movement from one xylem vessel to another in host plants. The same set of observations reports the absence of bacterial vessel occlusions in new branches of Cellina di Nardò plants with an asymptomatic canopy. In summary, these findings provide novel insights into the olive trees' adaptive response to *Xfp*.

## Graphical abstract

After long‐term infection, the susceptible *O. europaea* cultivar Cellina di Nardò modified its wood anatomical traits as an adaptive response to counteract *Xylella fastidiosa*, exhibiting spontaneous canopy restoration.

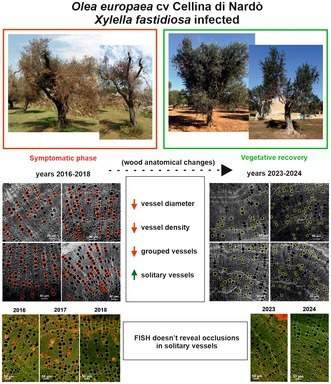

## INTRODUCTION

Since 2013, in the Salento peninsula (Apulia, South Italy), olive trees have been affected by the bacterium *Xylella fastidiosa* (Wells *et al*. 1987) subsp. *pauca* (*Xfp*) (Saponari *et al*. 2013), which is associated with olive quick decline syndrome (OQDS). *Xf* colonizes the xylem vessel network, developing bacterial aggregates with biofilm deposition and inducing, in the host plants, the production of tyloses and gum as active defence response to the invasion; these events, limiting water flow, cause drying out and the related symptoms development (Petit *et al*. 2021).

The olive tree (*Olea europaea* L. subsp. *europaea* var. *europaea*) is an evergreen tree species widely cultivated for its fruits and for olive oil production around the Mediterranean basin since the Roman empire (Caracuta 2020). Although olive trees are drought‐tolerant (Connor & Fereres 2004), dry conditions reduce their productivity, and therefore, several studies have been conducted to optimize performance. The ability of the xylem vessels to transport water is inextricably linked to water usage strategy and, as a consequence, to the net plant productivity in a given environment (Fonti & Jansen 2012). Therefore, studies of the anatomy of xylem tissue have gained considerable importance to understand how adaptive changes in wood anatomical structure and hydraulic architecture can be an efficient response to drought conditions (Fernandéz *et al*. 2011; Rossi *et al*. 2013). These studies showed how the xylem hydraulic architecture, with the vessel diameter, frequency, length, arrangement of conduits, etc., not only regulates hydraulic efficiency but also serves as a strategy for conductive safety, highlighting xylem plasticity in olive trees. So far, xylem plasticity/flexibility has been assessed mainly in relation to abiotic stress (Hoffmann *et al*. 2011; Carluccio *et al*. 2023). The Cellina di Nardò, a prevalent olive cultivar in Salento during the first European *Xfp* outbreak, was introduced by the Saracens in the 9th and 18th centuries (Caracuta 2020). When at the end of 1700 the ‘Brusca’ disease affected olive trees causing desiccations somewhat like that caused by *Xfp*, Cellina di Nardò proved to be ‘Brusca’‐resistant (Frisullo *et al*. 2014). On the contrary, since the beginning of the *Xfp* epidemiology, the cultivar appeared to be susceptible to the bacterium with rapid drying of the canopy. Anyway, and unexpectedly, more than 10 years after *Xfp* first report, some plants of Cellina di Nardò have shown a vegetative recovery, returning to being somehow productive with a fruit yield estimated by owners of 20–40% in comparison to pre‐*Xylella*. This phenomenon supports the hope of re‐establishing the typical olive oil production of the area in which, during the 3 years 2016–2018 when extensive Cellina di Nardò canopy desiccation was observed, the loss of olive production was estimated to be 29,000 tonnes (Frem *et al*. 2021). Other authors have previously reported this vegetative recovery: Scortichini & Ragno (2024) described this resurgence as ‘resilience’ monitoring the phenomenon on the two olive susceptible cultivars Cellina di Nardò and Ogliarola Salentina illustrating a survey in the Salento area; they noted that a ‘new crown was obtained upon the cutting of the main tree branches that wilted upon *Xylella fastidiosa* subsp. *pauca* infection and the sucker resprouting’ and enlisted predisposing factors without any hypothesis concerning the plant response. Literature, which has significantly increased in recent years, demonstrated that host response and the resistance mechanisms to *Xfp* have a multifactorial basis. Already in 2016, Giampetruzzi et al. (2016) reported a global quantitative transcriptome profiling of naturally infected plants and highlighted that susceptible and tolerant olive plants react to the *Xfp* infection by strong re‐modelling of cell wall proteins. Thereafter, Novelli *et al*. (2019) noticed in infected olive trees up‐regulation of defence‐related genes, such as NADPH oxidase, some protein kinases, pathogen plant response factors and metabolic enzymes, and in Leccino plants an enhanced production of specific antioxidant and antimicrobial molecules, to fight the pathogen. Consequently, La Notte *et al*. (2024) analysed differentially expressed genes in olive trees and concluded that a plant defence response occurs in *Xfp*‐infected tissues, specifically that molecular functions relying on the activity of cell wall‐associated protein kinases involved in the basal immunity response are enhanced in *Xfp*‐infected tissues. Just recently, Abou Kubaa *et al*. (2026) evaluated the transcriptional response in clones of the resistant olive cultivar Leccino inoculated with *Xfp*; they found differentially expressed genes involved in plant immunity and, among these genes, cell wall‐related genes, suggesting that cell wall dynamics have a decisive role in plant immunity functioning both as a stage for pathogen recognition and as a physical defence. In fact, cell wall patterning leads to xylem differentiation and determines the arrangement of xylem vessels to ensure, first of all, proper water transport. In our work, conscious of the olive xylem plasticity, we aimed to identify how plants respond to *Xylella* and to the environment focusing our attention on the anatomical traits that usually are at the base of the olive drought tolerance, investigating whether an anatomical adaptation may contribute to the survival of Cellina di Nardò plants, a variety susceptible to *Xfp*, in an infected area.

## MATERIALS AND METHODS

### Study sites and sample collection

**Fig. 1:**
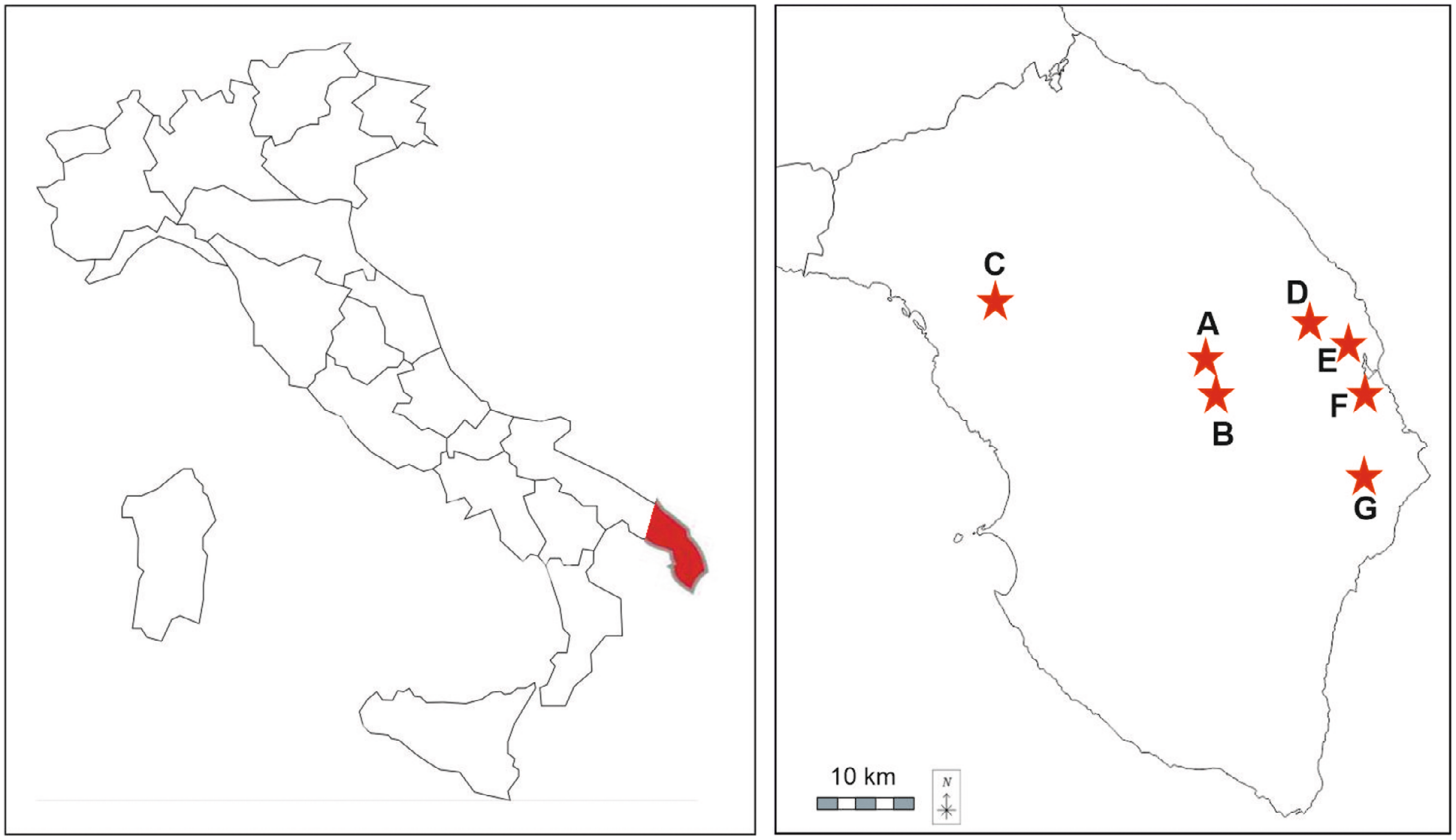
Sampled sites located in Salento areas (Apulia region) where *X. fastidiosa* is considered endemic and where Cellina di Nardò olive trees were hosted. Sites A and B are in the municipality of Martano; site C is in Leverano; site D is located in Melendugno; sites E and F are close to Alimini lakes (municipality of Otranto); site G is in Otranto.

**Fig. 2:**
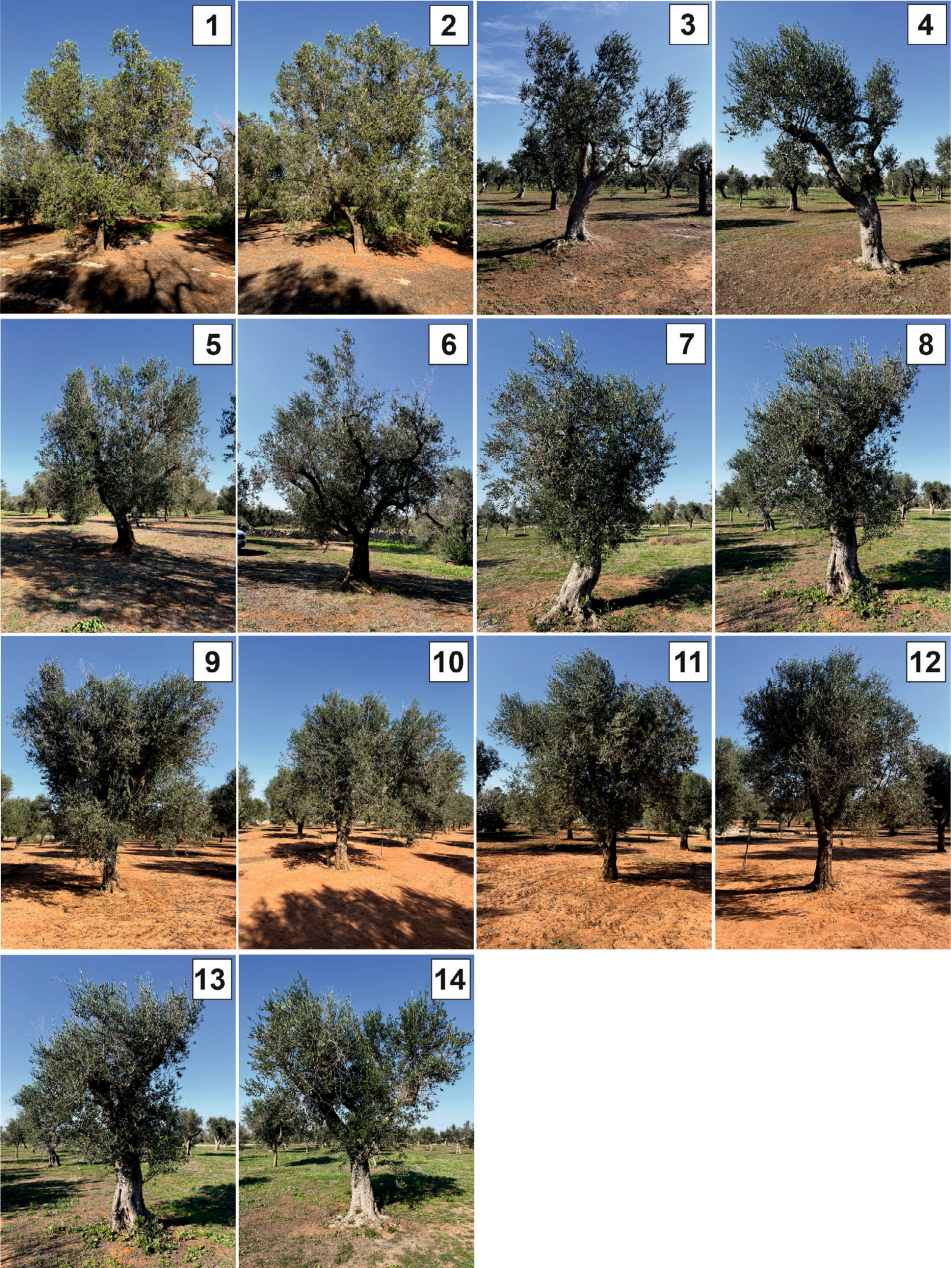
Representative phenotypes of Cellina di Nardò olive plants studied in this work. Plants are located in the sampling sites as follows: plants 1 and 2 in site A, Martano; plants 3 and 4 in site B, Martano; plants 5 and 6 in site C, Leverano; plants 7 and 8 in site D, Melendugno; plants 9 and 10 in site E, Alimini; plants 11 and 12 in site F, Alimini; plants 13 and 14 in site G, Otranto.

Seven olive (*Olea europaea* L.) orchards were chosen (Fig. 1) in the Salento area, Lecce province (Apulia, South Italy), an area delimited as *Xf‐*infected in 2015 (BURP‐ Bollettino Ufficiale della Regione Puglia n. 15 del 29‐01‐2015, n.d.) following the EU Commission Implementing Decision of 13 February 2014 (EU Commission, n.d.‐a). These orchards are composed of the cultivar Cellina di Nardò, susceptible to the *Xfp*, and they were chosen because in the last 3 years (2022–2024) these olive trees showed an evident vegetative recovery (Fig. 2).

Selected plants received the same agronomic practices and vector insect control over 8 years according to EU Commission Implementing Decision of 18 May 2015 (2015/789/EU); furthermore, examined plants were monitored and tested for some of the most common pathogens, including *Colletotrichum gloeosporioides*, *Mycocentrospora cladosporioides*, *Spilocaea oleagina* and *Pseudomonas savastanoi* pv. *savastanoi* (EU Commission, n.d.‐b). Diagnostic tests (real‐time PCR) were carried out on leaves or woody chips, according to protocols reported in the literature, for *Verticillium dahliae* (Bilodeau *et al*. 2012), *Colletotrichum* spp., *C. acutatum* and *C*. *gloeosporioides* (Garrido *et al*. 2009), *Phaeomoniella chlamydospora* (Martin *et al*. 2012), *Phaeoacremonium aleophilum* and *Phaeoacremonium parasiticum* (Aroca *et al*. 2008; Carlucci *et al*. 2015), *Botryosphaeria dothidea* (Romanazzi *et al*. 2009), *Diplodia seriata* (Martin *et al*. 2014) and *Phytophthora* spp. (Drenth *et al*. 2006). Apart from some sporadic evidence of *C*. *gloeosporioides*, *S*. *oleagina* and *P*. *savastanoi* pv. *savastanoi*, trees tested negative for other pathogens.

In accordance with the Guidelines for the survey of *Xylella fastidiosa* in the EU territory (2015/789/EU), indicating that plant surveys and sampling in open fields should occur during the vegetative season, all plant material was collected in July across the specified years below (EU Commission, n.d.‐c). A total of 21 plants (three each site) were sampled. The same plants (three plants per orchard) were sampled during the symptomatic phase (years 2016–2018) and the vegetative recovery phase (years 2023–2024). In each sampling campaign, eight twigs (1‐year‐old) from each plant were collected, representative of the entire aerial part of the plant and utilized for analyses. The infected wood samples collected during the symptomatic and the vegetative recovery phase were compared with 1‐year‐old branches from healthy Cellina di Nardò plants sampled in the years 2014–2015, before the destructive *Xylella fastidiosa* subsp. *pauca* epidemic spread, from olive groves located in the same area and therefore subject to similar soil and climate conditions compared with fields A–F. Furthermore, to include negative control samples from a *Xfp*‐free area, three healthy Cellina di Nardò plants (over 26 years old) were collected in December 2025 from the research centre of Mirto – Crosia (Cosenza, Italy) where the CREA‐OFA (Council for Agricultural Research and Economics – Research Centre for Olive, Fruit and Citrus Crops) manages a collection of more than 600 varieties of olive trees, including 53 foreign varieties; this comparison was due to the unavailability of healthy and non‐infected Cellina di Nardò plants in the *Xfp*‐infected area, while greenhouse control plants are not applicable due to significant age discrepancies with field plants. Samples from healthy plants underwent identical treatment as those from infected ones, including the monitoring and testing for some of the most common pathogens.

### Detection of *X. fastidiosa* and NDVI (normalized difference vegetation index) assessment

Diagnostic analyses were conducted to evaluate or verify the presence of *Xfp* in the control healthy plants and in the infected plants during the two sampling campaigns (2016–2018 and 2023–2024). The pooled samples (1 g for each plant) used for *Xfp* detection were randomly taken from eight twigs sampled from each tree. DNA extraction was carried out by using the CTAB (cetyltrimethyl ammonium bromide) method (Loconsole *et al*. 2014). The DNA was used as template for *Xfp* detection by TaqMan real‐time PCR protocol with XF‐F and XF‐R primers, and with XF‐P probe proposed by Harper *et al*. (2010). The reactions were performed in a Real‐Time thermal cycler (QuantStudio 3 Real‐Time PCR System, Applied Biosystem, Foster City, USA) in a final volume of 20 μl containing 10 μl of TaqMan Fast Advanced Master Mix (Applied Biosystem, Foster City, USA), 300 nM of XF‐F and XF‐F primers, 100 nM of XF‐P probe, ultrapure DNase/Rnase‐free water (Carlo Erba Reagents S.r.l., Milan, Italy) and 2 μl of DNA (20 ng μl^−1^). The cycling conditions were as follows: initial uracil–DNA glycosylase incubation at 50 °C for 2 min, polymerase activation at 95 °C for 20 s, followed by 40 cycles at 95 °C for 1 s and 60 °C for 20 s. The *Xfp* concentration, expressed as bacterial cfu ml^−1^, was determined as described by D'Attoma *et al*. (2019) from the cycle of quantification (Cq) values of dilutions ranging from 10^2^ to 10^7^ cfu ml^−1^. To compare canopy vegetation changes between the years 2016–2018 and 2023–2024, we have used the normalized difference vegetation index (NDVI) which is a widely used metric for quantifying the health and density of vegetation (Vanella *et al*. 2020). In this study, NDVI time‐series, referring to sampled fields A–G, were analysed in the periods from 1 January 2016 to 31 December 2018 and from 1 January 2023 to 31 December 2024. Data of the vegetation index were extracted and downloaded from the Global Subsets Tool (Vannan *et al*. 2009). This tool (https://modis.ornl.gov/cgi‐bin/MODIS/global/subset.pl) provides MODIS products that can be freely used by the community for several employments (*e.g*., for validating model, for characterizing field sites, etc.). Specifically, Terra‐MODIS vegetation index (VI) product (MOD13Q1, Version 6 Level 3) was used for the time‐series analyses (Didan 2015). This index oscillates between +1 and −1, where the higher value indicates greener and denser vegetation while NDVI close to 0 or negative NDVI identifies non‐vegetated zones (Rouse *et al*. 1973).

### Xylem anatomical measurements: Vessel diameter, frequency and cavitation vulnerability index

Xylem anatomical measurements were performed on cross‐sections of the sampled 1‐year‐old branches (eight twigs from each plant were collected; the same three plants for orchards were analysed) in the healthy control plants and in the infected plants both during the symptomatic phase (years 2016–2018) and during the years of vegetative recovery (2023–2024). 1‐year‐old twigs were chosen to study wood anatomical features both because the owners do not allow the plants to be cut down for analysis of the trunk and because, according to De Micco *et al*. (2008), the adaptive trends in qualitative/quantitative anatomical wood features are equally expressed in twig and stemwood in response to habit water availability. One‐year‐old twigs were cut into pieces approximately 6 mm long; every cut piece was dehydrated with increasing ethanol concentration series and embedded in paraffin employing an automatic Leica TP 1020 (Leica Microsystems, Mannheim, Germany) tissue processor. The paraffin‐included branch pieces were cut into 25 μm thick cross‐sections with the HistoCore MULTICUT R semi‐automatic rotary microtome (Leica Microsystems, Mannheim, Germany). Obtained sections were then transferred to a 1:1 (v/v) PBS: 96% ethanol solution and stored at −20 °C until staining. To remove paraffin, sections were embedded for 3 min at 43 °C in toluene, which was just after discarded. Thus, all sections were stained with a safranin solution (1%) to enhance contrast of wood against void space of vessels (Sabella *et al*. 2019), and then fixed with Canada balsam. All images were taken on a confocal laser scanning microscope (Carl Zeiss LSM 700 laser scanning microscope, Jena, Germany) and acquired with the same microscope settings. All images were analysed using ImageJ v1.46r. The vessel diameter and the vessel distribution (number of vessels per mm^2^, N/mm^2^) were computed according to Scholz *et al*. (2013). Ten sections from every branch piece were analysed (a total of 80 sections per plant). The vulnerability to cavitation (vulnerability index, VI) was determined following the definition of vulnerability (V) made by Carlquist (1977) as follows:

(1) VI=VD/VF

where VD is the mean vessel diameter (μm), and VF is the vessel density/frequency (number of vessels per mm^2^)

### Vessel arrangement: Vessel grouping indices (V_G_ , V_S_ and V_M_ )

The vessel grouping index (V_G_) defined by Carlquist (2001) corresponds to the total number of vessels divided by the total number of vessel groupings, which is the sum of solitary vessels (counting as one vessel group) plus vessel clusters and radial multiples. The V_G_ does not take into account the diameter of vessels, and a value of 1 indicates the exclusive presence of solitary vessels; the higher the index, the greater the degree of vessel grouping/vascular clustering. V_G_ is the opposite of the vessel multiple index (V_M_), which represents the ratio between the number of vessel groupings and the total number of xylem vessels. The solitary vessel index (V_S_) is defined as the ratio of the total number of solitary vessels to the total number of solitary and grouped vessels.

Table S1 summarizes the three indices that were defined to quantify vessel groupings (Spannl *et al*. 2016).

### Fluorescence *in situ* hybridization

Sections, obtained as reported above in the method description for the xylem anatomical measurements, were processed according to Cardinale *et al*. (2018). In brief, sections were permeabilized with 0.5 mg ml^−1^ lysozyme (Life Technologies Italia, Milan, Italy) for 10 min at room temperature after removing toluene and two 5‐min rinses in PBS buffer, and following dehydration (ethanol 96–70–50%, 3 min each), hybridization was carried out at 42 °C for 120 min with the Cy3‐labelled *X. fastidiosa*‐specific KO 210 probe (Cardinale *et al*. 2018). A Citifluor AF1 antifade reagent (Linaris Biologische Produkte GmbH, Dossenheim, Germany) was used for the montage onto the glass slide. The slides could be stored for up to 4 days in the dark at 4 °C. A confocal laser‐scanning microscope (Carl Zeiss LSM 700, Jena, Germany) was used for the observations. Cy3 was excited with the laser line 561 nm; the laser line 405 was used to induce the autofluorescence of tissues. Emissions were captured in the range of 570–613 nm for Cy3 and 420–480 nm for the plant autofluorescence.

### Statistical analysis

All data were statistically analysed using two‐way ANOVA followed by Tukey‐HSD (honestly significant difference) *post hoc* test (*P* < 0.05). Analyses were achieved using R version 3.5.3.

## RESULTS

### *Xylella fastidiosa* detection and normalized difference vegetation index (NDVI)

The diagnostic real‐time PCR tests confirmed the presence of the *Xfp* infection in all the sampled plants of Cellina di Nardò both during the symptomatic phase (years 2016–2018) and during the years of vegetative recovery (Table S2). The bacterial concentrations detected in one‐year‐old branches were relatively high during the two periods, with averages of 4.46 × 10^5^, 1.71 × 10^6^, 1.85 × 10^7^, 2.03 × 10^5^ and 2.49 × 10^6^ cfu ml^−1^ respectively in 2016, 2017, 2018, 2023 and 2024 (Table S2).

**Fig. 3:**
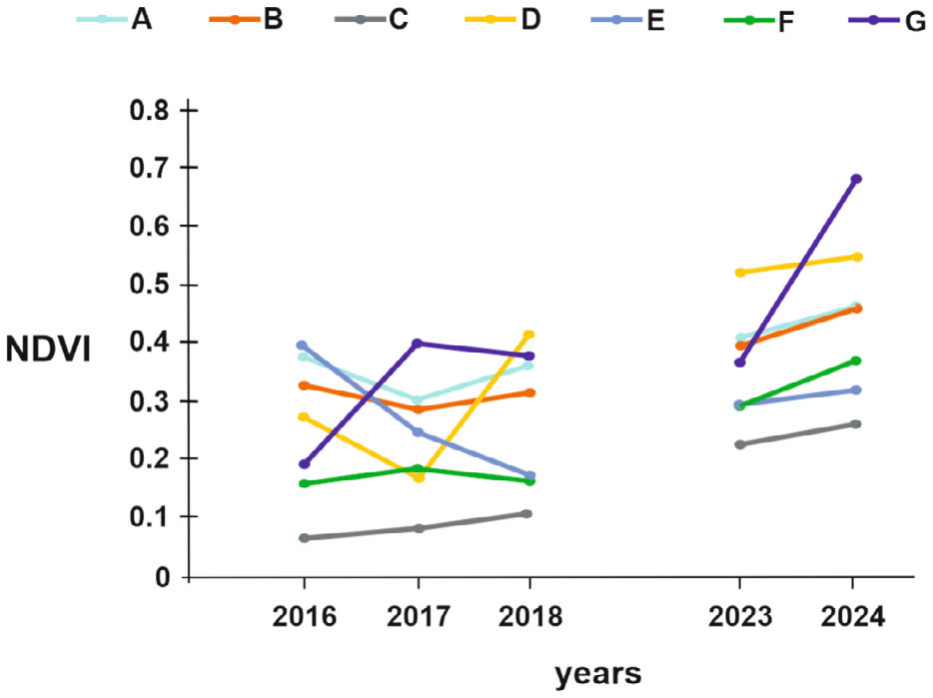
Normalized difference vegetation index (NDVI) for the years 2016–2018 and 2023–2024, in the seven sampling sites (named from A to G).

The NDVI values calculated for the seven Cellina di Nardò olive orchards (named from A to G) showed a positive trend from 2016 to 2024 (Fig. 3). In site A, NDVI values ranged from 0.28 to 0.45; in site B from 0.28 to 0.44; from 0.06 to 0.25 in C; from 0.27 to 0.54 in D; from 0.16 to 0.31 in E; from 0.16 to 0.35 in F; and finally, in G from 0.17 to 0.68.

The NDVI data indicated a vegetative recovery of the Cellina di Nardò canopy in the examined orchards that can be also appreciated by the photointerpretation of the Google Earth (https://earth.google.com/web/) satellite images of the A–G sites (Fig. S1). This regrowth can be also observed in many other infected fields of the area.

### Wood anatomical traits in *Xfp* ‐infected Cellina di Nardò with canopy vegetative recovery

**Fig. 4:**
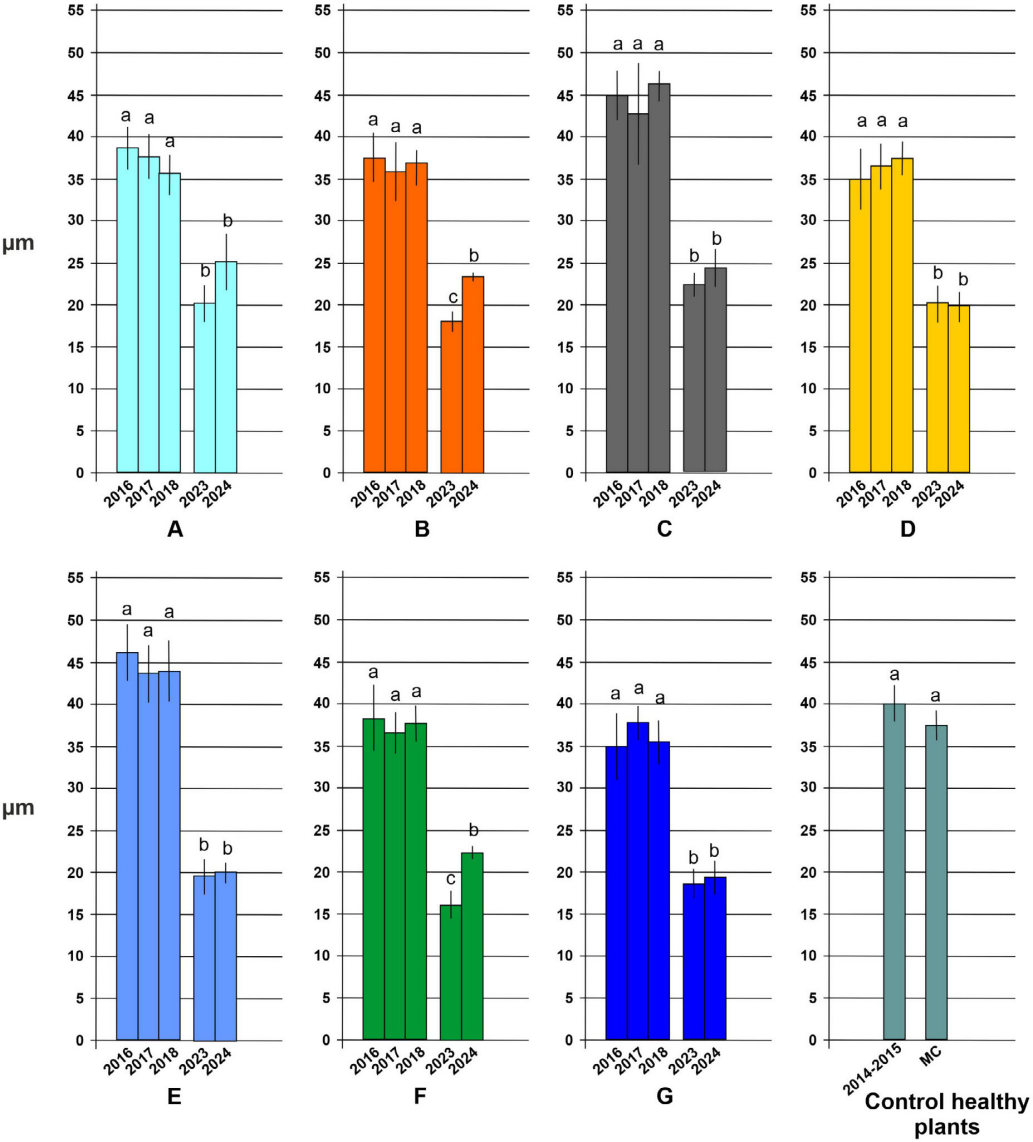
Xylem vessel diameter (μm) in 1 year‐old branch of Cellina di Nardò olive trees sampled in 2023 and 2024 compared with diameter of the same samples collected in the years 2016, 2017 and 2018 in the seven sampling sites (named from A to G). Control healthy plants: healthy plants sampled in the years 2014–2015 before the *Xfp* epidemic spread (2014–2015); plants sampled in the *Xfp*‐free area of Mirto‐Crosia, Cosenza, Italy (MC). Columns represent average values of three tree samples (eight twigs per plant, 10 sections from every twig, for a total number of 80 sections per plant). Statistical analysis was performed through ANOVA (*P*‐value <0.05), followed by Tukey‐HSD *post hoc* test. Different letters correspond to statistically different means.

To investigate if modifications of wood anatomical traits of Cellina di Nardò may have played a role in the observed vegetative recovery, we compared the xylem vessel diameter of 1 year‐old branches sampled during canopy vegetative recovery in 2023 and 2024 to those sampled in the years 2016–2018 when the same plants showed severe desiccation symptoms. The data obtained indicated a considerable diameter reduction in the years 2023–2024 in all the seven studied orchards (Fig. 4). The average xylem vessel diameter characterizing the symptomatic Cellina di Nardò 1‐year‐old branches in the years 2016, 2017 and 2018 were respectively 39.26 ± 4.76, 37.89 ± 4.63 and 38.16 ± 5.24 μm; the average diameter measured in Cellina di Nardò twigs in vegetative recovery in 2023 and 2024 ranged from the minimum value 16.18 ± 1.68 μm (orchard F sampled in the 2023) to the highest value 25.16 ± 3.34 μm (orchard A sampled in 2024). The average diameter of the xylem vessels during vegetative recovery is significantly (*P*‐value = 0.000196) lower than the average diameter measured in healthy Cellina di Nardò plants sampled before the spread of the epidemic (years 2014–2015); this value, corresponding on average to 40.07 ± 2.35 μm, does not show a statistically significant (*P*‐value = 0.069169) difference compared with the average diameter recorded in symptomatic infected plants. The data were also confirmed by the comparison with the healthy Cellina di Nardò controls sampled in disease‐free area: the average measured diameter of the xylem vessels is 37.44 ± 1.96 μm; it is significantly (*P*‐value = 0.000213) greater if compared with plants in vegetative recovery; nevertheless, there is no statistically significant (*P*‐value = 0.078225) difference with the average diameter of the symptomatic infected plants.

**Fig. 5:**
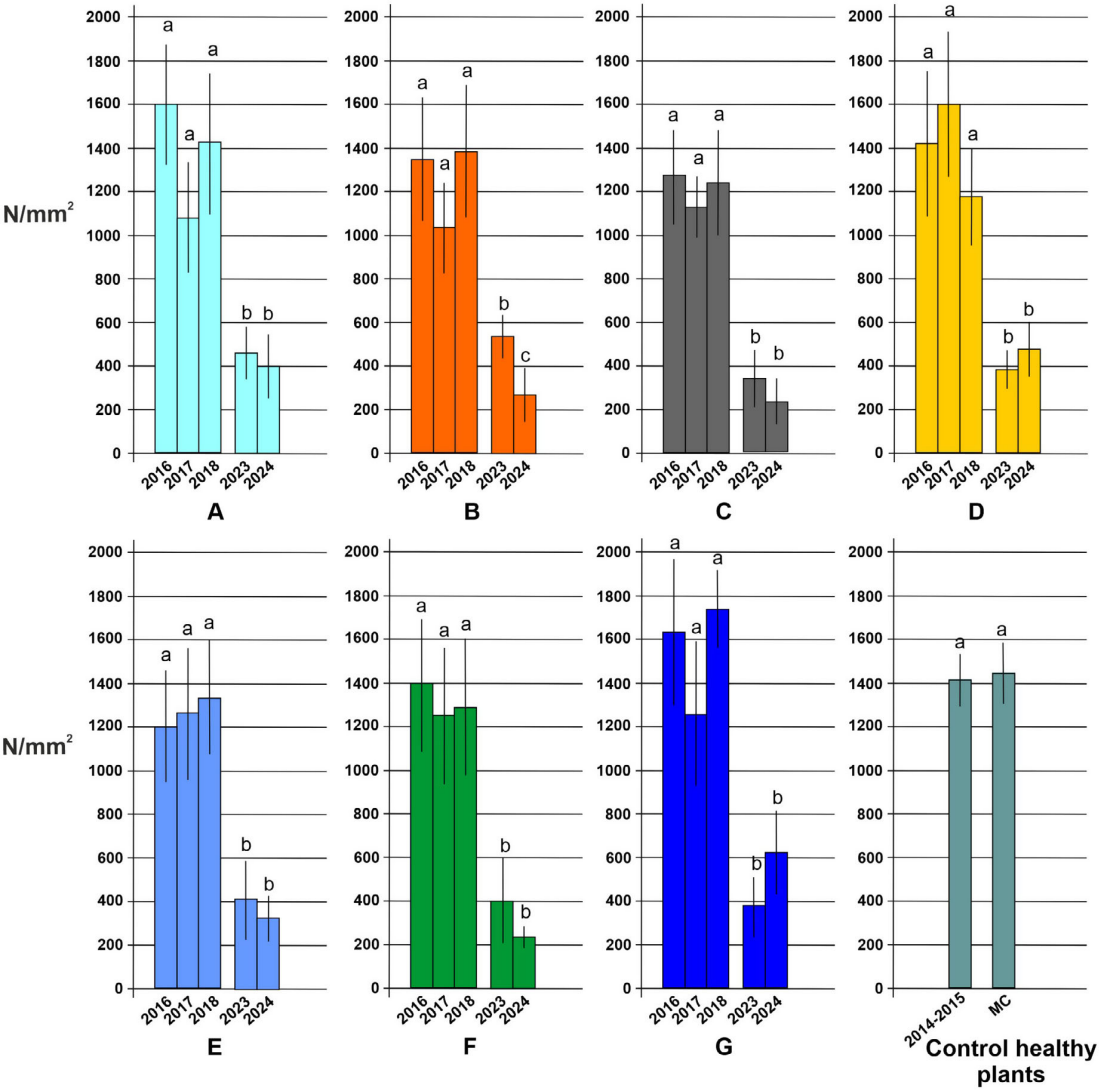
Xylem vessel density (N/mm^2^) in 1‐year‐old branch of Cellina di Nardò olive trees sampled in 2023 and 2024 compared with cavitation vulnerability of the same samples collected in the years 2016, 2017 and 2018 in the seven sampling sites (named from A to G). Control healthy plants: healthy plants sampled in the years 2014–2015 before the *Xfp* epidemic spread (2014–2015); plants sampled in the *Xfp*‐free area of Mirto‐Crosia, Cosenza, Italy (MC). Columns represent average values of three tree samples (eight twigs per plant, 10 sections from every twig, for a total number of 80 sections per plant). Statistical analysis was performed through ANOVA (*P*‐value <0.05) followed by Tukey‐HSD *post hoc* test. Different letters correspond to statistically different means.

Also, Cellina di Nardò 1‐year‐old branches sampled in 2023/2024 had significantly lower xylem vessel density/frequency (number of vessels per mm^2^) than symptomatic plants from 2016 to 2018 in all the seven field sites (Fig. 5). In fact, in 2016–2018 the average density of the vessels was 1407.95 ± 157.07, 1212.94 ± 201.96 and 1359.20 ± 191.69 vessels per mm^2^, respectively, while in plants sampled in 2023/2024 the values ranged between 217.91 (orchard C sampled in 2024) and 598.86 vessels per mm^2^ (orchard G sampled in 2024) (Fig. 5). In the healthy Cellina di Nardò plants, sampled between 2014 and 2015, the average vessel frequency (1417.67 ± 199.32) was statistically similar (*P*‐value = 0.084133) to the values measured in symptomatic plants; statistically similar (*P*‐value = 0.079568) to these plants were also the healthy Cellina di Nardò controls from disease‐free area with a vessel density of 1436.88 ± 142.10 vessels per mm^2^.

**Fig. 6:**
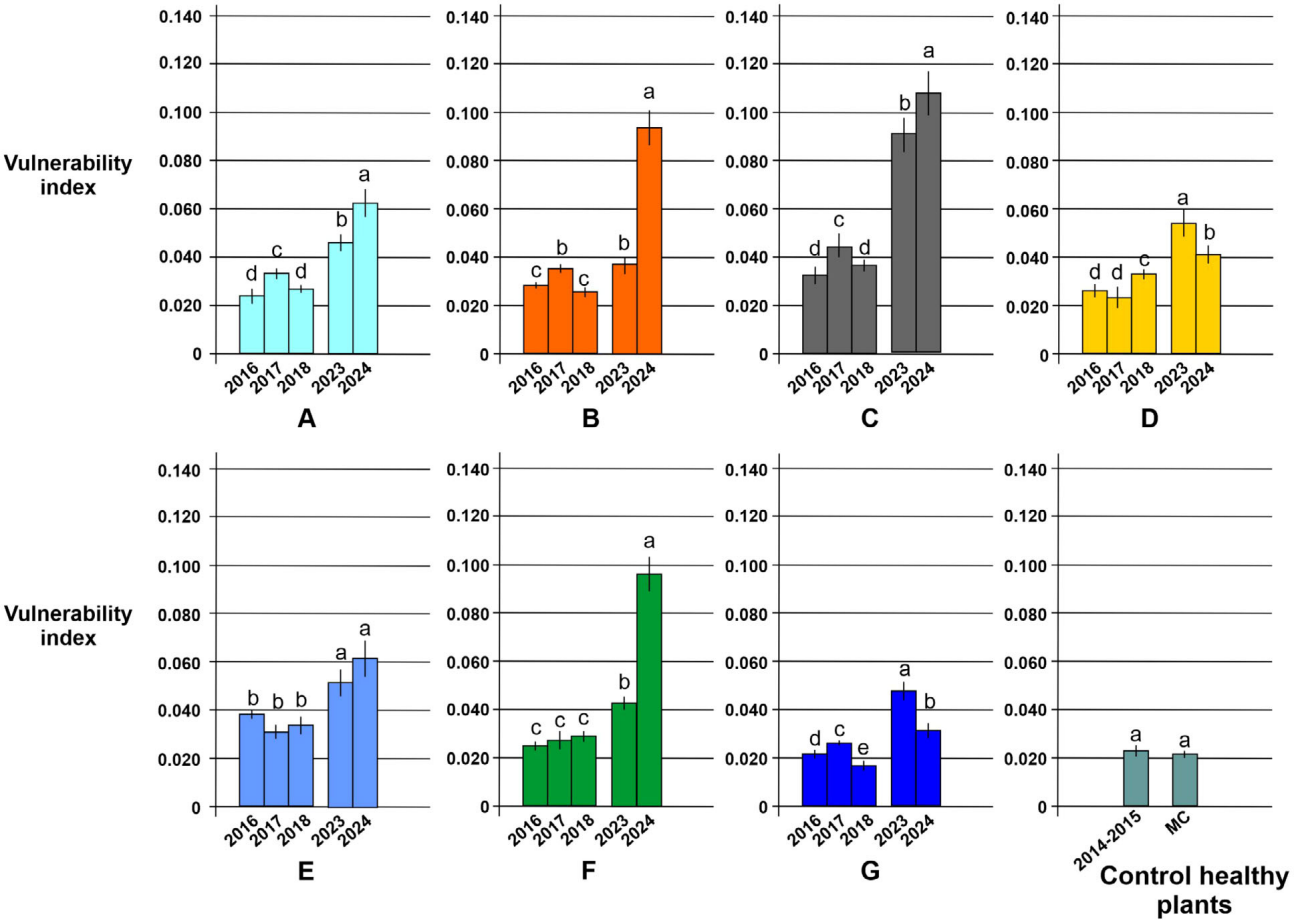
Vulnerability index to cavitation in 1‐year‐old branch of Cellina di Nardò olive trees sampled in 2023 and 2024 compared with cavitation vulnerability of the same samples collected in the years 2016, 2017 and 2018 in the seven sampling sites (named from A to G). Control healthy plants: healthy plants sampled in the years 2014–2015 before the *Xfp* epidemic spread (2014–2015); plants sampled in the *Xfp*‐free area of Mirto‐Crosia, Cosenza, Italy (MC). Columns represent average values of three tree samples (eight twigs per plant, 10 sections from every twig, for a total number of 80 sections per plant). Statistical analysis was performed through ANOVA (*P*‐value <0.05) followed by Tukey‐HSD *post hoc* test. Different letters correspond to statistically different means.

As a direct consequence of the variations recorded in the average vessel diameter and, specifically, in the vessel frequency in 2023/2024, the index of vulnerability to cavitation tended to significantly increase (Fig. 6), while it remains practically similar to that of 2016–2018 plants for the 2014–2015 healthy control plants and for the Mirto‐ Crosia samples (Fig. 6).

### A xylem vessel re‐arrangement in susceptible cultivar Cellina di Nardò after long‐term *Xylella fastidiosa* infection

**Fig. 7:**
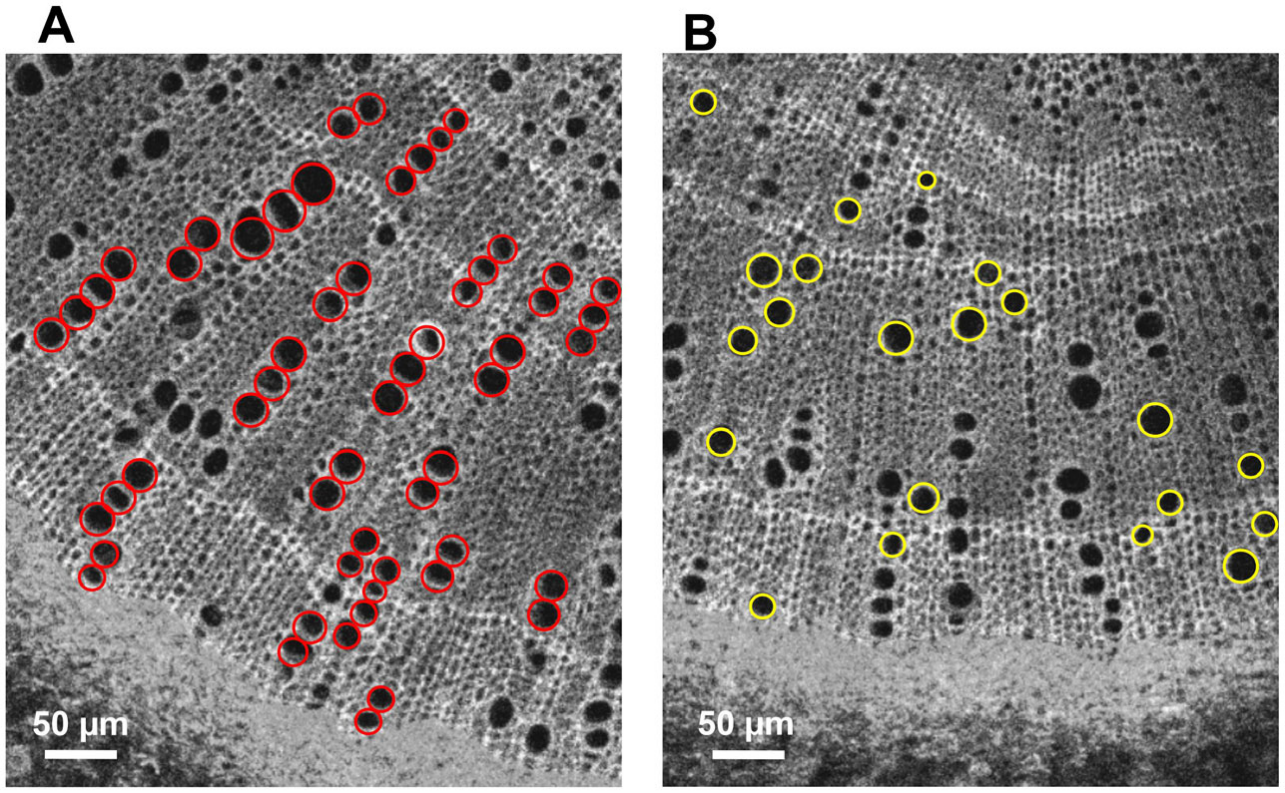
Representative images of the vessel re‐arrangement found in 1 year‐old branch. (A) symptomatic Cellina di Nardò sampled in 2016–2018; (B) Cellina di Nardò plant in vegetative recovery sampled in 2023–2024. Red circles highlight grouped vessels; yellow circles mark solitary vessels.

The vessel re‐arrangement was evaluated through three indices: the V_G_, the vessel solitary index (V_S_) and the V_M_ (Tables 1, 2, 3). Compared with symptomatic plants in 2016–2018, 1‐year‐old branches of plants examined in 2023/2024 with canopy restoration showed substantially lower levels of V_G_ (Table 1). These data indicated a trend in the plants of 2023/2024 to reduce the vessel grouping and to enhance solitary vessels. In fact, the obtained vessel solitary indexes (V_S_, Table 2) in 1‐year‐old branches sampled in 2023–2024 described a significant increase in solitary vessels in contrast with the previously considered years (Fig. 7; Fig. S2).

**Table 1:** Data show vessel grouping index (V_G_ = N_*vessels*_/N_*groupings*_) in 1 year‐old branches of the same infected Cellina di Nardò olive trees during the symptomatic phase in the years 2016–2018, during the vegetative recovery in 2023–2024 for the seven sampling sites (A–G), of healthy Cellina di Nardò plants sampled in the years 2014 and 2015 (before the *Xfp* spread), and in 2025 in the disease‐free area of Mirto – Crosia (Cosenza, Italy). Statistical analysis was performed through ANOVA (*P*‐value <0.05) followed by Tukey‐HSD *post hoc* test.

	Vessel grouping index (V_G_)						
	2016	2017	2018	2023	2024	Healthy (2014–2015)	Healthy (Mirto‐Crosia 2025)
A	2.96 ± 0.21a	2.61 ± 0.12ab	2.79 ± 0.14ab	2.03 ± 0.20cd	1.73 ± 0.08e		
B	2.83 ± 0.19ab	2.90 ± 0.16a	2.64 ± 0.17ab	2.21 ± 0.16c	1.55 ± 0.11ef		
C	2.74 ± 0.08ab	2.87 ± 0.10a	2.76 ± 0.11ab	1.68 ± 0.07e	1.53 ± 0.04f		
D	2.73 ± 0.08ab	2.76 ± 0.12ab	2.75 ± 0.09ab	1.94 ± 0.02d	1.75 ± 0.14de	2.84 ± 0.16a	2.93 ± 0.19a
E	2.92 ± 0.17a	2.79 ± 0.15ab	2.57 ± 0.08b	1.98 ± 0.23cd	1.62 ± 0.14ef		
F	2.72 ± 0.08ab	2.84 ± 0.10a	2.73 ± 0.09ab	1.67 ± 0.17de	1.46 ± 0.07f		
G	2.94 ± 0.11a	2.79 ± 0.14ab	2.86 ± 0.10a	1.87 ± 0.09de	1.57 ± 0.04ef		

Different letters correspond to statistically different means.

**Table 2:** Data show vessel solitary index (V_S_ = N_*solitaryvessels*_/N_*vessels*_) in 1 year‐old branches of the same Cellina di Nardò olive trees during the symptomatic phase in the years 2016–2018, during the vegetative recovery in 2023–2024 for the seven sampling sites (A–G), of healthy Cellina di Nardò plants sampled in the years 2014 and 2015 (before the *Xfp* spread), and in 2025 in the disease‐free area of Mirto‐Crosia (Cosenza, Italy). Statistical analysis was performed through ANOVA (*P*‐value <0.05) followed by Tukey‐HSD *post hoc* test.

	Vessel solitary index (V_S_)						
	2016	2017	2018	2023	2024	Healthy (2014–2015)	Healthy (Mirto‐Crosia 2025)
A	0.03 ± 0.01f	0.11 ± 0.01e	0.07 ± 0.02ef	0.19 ± 0.06ce	0.31 ± 0.05bc		
B	0.06 ± 0.02ef	0.04 ± 0.01f	0.06 ± 0.03ef	0.13 ± 0.04e	0.37 ± 0.08ab		
C	0.08 ± 0.03ef	0.10 ± 0.04 ef	0.07 ± 0.03ef	0.32 ± 0.04b	0.37 ± 0.06ab		
D	0.11 ± 0.04ef	0.08 ± 0.03 ef	0.09 ± 0.04ef	0.19 ± 0.01d	0.30 ± 0.07bc	0.09 ± 0.03ef	0.11 ± 0.02ef
E	0.09 ± 0.02ef	0.07 ± 0.01f	0.07 ± 0.01f	0.18 ± 0.04ce	0.25 ± 0.03c		
F	0.07 ± 0.02ef	0.08 ± 0.01ef	0.10 ± 0.01ef	0.30 ± 0.07bc	0.44 ± 0.05a		
G	0.04 ± 0.01f	0.11 ± 0.02e	0.06 ± 0.04ef	0.22 ± 0.03cd	0.37 ± 0.02ab		

Different letters correspond to statistically different means.

The third calculated index, vessel multiple index (V_M_, Table 3) did not reveal significant differences among the analysed samples indicating that the multiple groups, on average, consisted of the same number of vessels ranged among 2.25 ± 0.16 and 2.69 ± 0.05 (Table 3).

**Table 3:** Data show vessel multiple index (V_M_ = N_*multiplevessels*_/N_*multiplegroupings*_) in 1‐year‐old branches of the same Cellina di Nardò olive trees during the symptomatic phase in the years 2016–2018, during the vegetative recovery in 2023–2024 for the seven sampling sites (A–G), of healthy Cellina di Nardò plants sampled in the years 2014 and 2015 (before the *Xfp* spread), and in 2025 in the disease‐free area of Mirto‐Crosia (Cosenza, Italy). Statistical analysis was performed through ANOVA (*P*‐value <0.05) followed by Tukey‐HSD *post hoc* test.

	Vessel multiple index (V_M_)						
	2016	2017	2018	2023	2024	Healthy (2014–2015)	Healthy (Mirto‐Crosia 2025)
A	2.35 ± 0.24a	2.44 ± 0.26a	2.65 ± 0.14a	2.66 ± 0.07a	2.55 ± 0.09a		
B	2.51 ± 0.11a	2.48 ± 0.20a	2.61 ± 0.31a	2.69 ± 0.05a	2.29 ± 0.15a		
C	2.27 ± 0.08a	2.39 ± 0.28a	2.56 ± 0.31a	2.45 ± 0.01a	2.25 ± 0.16a		
D	2.33 ± 0.17a	2.53 ± 0.19a	2.47 ± 0.07a	2.51 ± 0.01a	2.54 ± 0.06a	2.52 ± 0.16a	2.53 ± 0.18a
E	2.52 ± 0.13a	2.29 ± 0.16a	2.43 ± 0.21a	2.51 ± 0.26a	2.36 ± 0.10a		
F	2.61 ± 0.29a	2.42 ± 0.05a	2.35 ± 0.09a	2.45 ± 0.35a	2.28 ± 0.08a		
G	2.54 ± 0.12a	2.51 ± 0.09a	2.48 ± 0.07a	2.49 ± 0.13a	2.35 ± 0.10a		

Different letters correspond to statistically different means.

The vessel organization detected in the infected plants during the symptomatic phase and the vegetative recovery phase was compared with the average data measured in the healthy Cellina di Nardò plants (sampled in the Salento area in 2014–2015 and from the olive tree collection in the disease‐free area of Mirto‐Crosia, Cosenza, Italy, in December 2025) highlighting that during the symptomatic phase (years 2016–2018) the vessel spatial organization was statistically similar to those of the healthy plants (Tables 1, 2, 3; Fig. S3; Fig. S4).

### Xylem vessel re‐arrangement affects pattern of *X. fastidiosa* distribution in plant

**Fig. 8:**
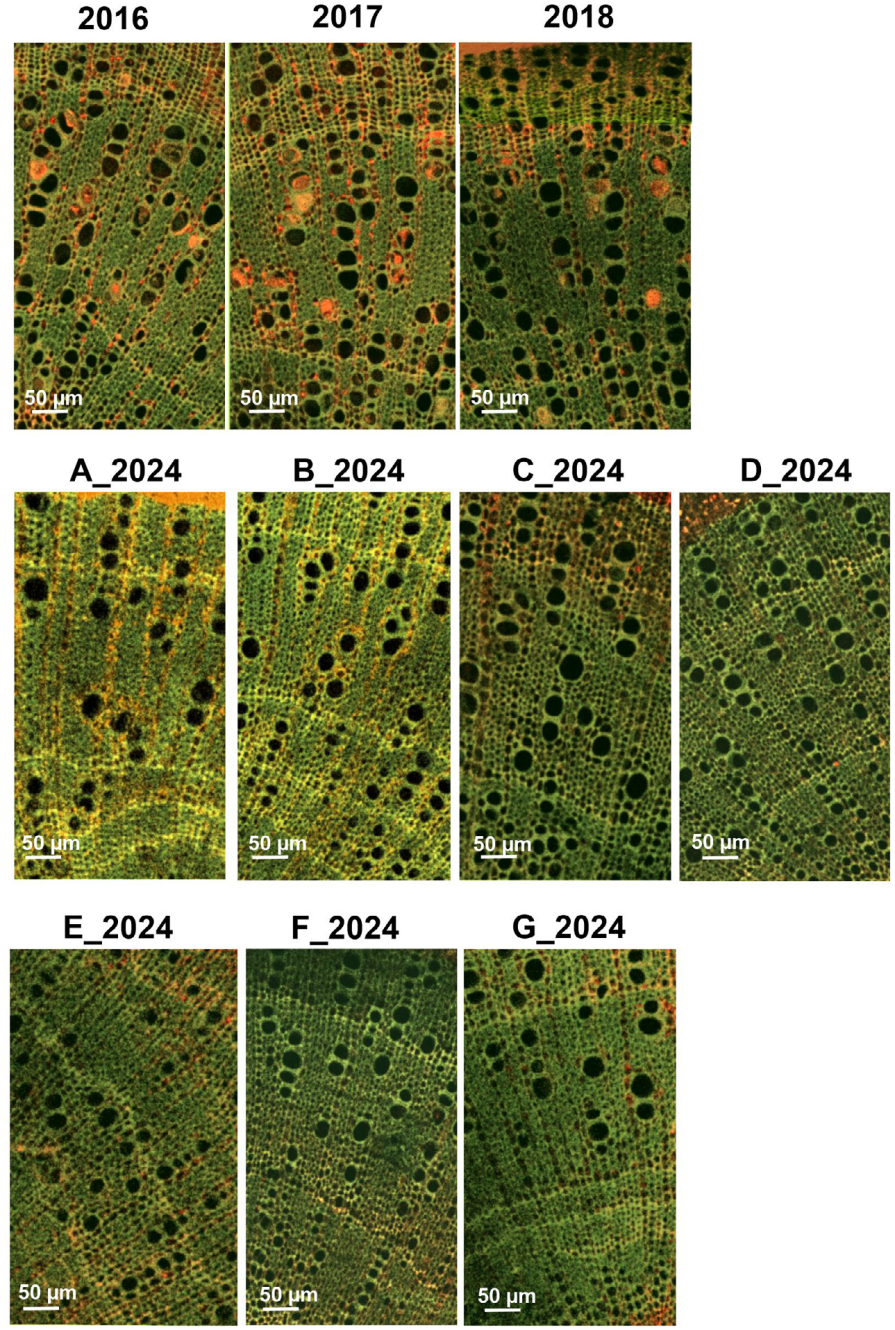
Representative images of xylem vessels subjected to FISH staining (Fluorescence *In Situ* Hybridization) with the KO 210 probe specific for *Xylella fastidiosa*. Red signal: probe specific for *Xfp* labelled with the dye Cy3. Images named as 2016, 2017 and 2018 represents the 1‐year‐old twig vessels of symptomatic Cellina di Nardò plants sampled in years 2016–2018. Images indicated as A_2024, B_2024, C_2024, D_2024, E_2024, F_2024 and G_2024 showed the xylem vessels in Cellina di Nardò plants in vegetative recovery sampled in 2023–2024 in each orchards site (A–G).

To evaluate the effect of vessel arrangement on the *Xfp* pattern distribution, bacteria were labelled with the *Xfp* specific probe KO 210 (Cardinale *et al*. 2018). The representative images of symptomatic plant vessels of 2016–2018 revealed a typical vessel occlusion of the entire vessel lumen (Fig. 8). These occlusions can be recognized mostly in the vessels organized in groups and, thus, having a substantial intervessel connection. On the contrary, in the vessel of the Cellina di Nardò plants in vegetative recovery of the canopy, sampled in 2023–2024, no significant occlusions were observed (Fig. 8).

## DISCUSSION

In this work, we examined changes in xylem anatomical traits of Cellina di Nardò olive trees to better understand their response to infection over time. Vessel diameter and density were among the analysed anatomical traits. The decrease in the average xylem vessel diameter in plants sampled in 2023–2024 is consistent with previously published papers that evaluated the impact of xylem geometry on olive cultivar capacity to outcome the pathogen, providing additional evidence that resistant cultivars are characterized by narrower xylem vessels (Sabella *et al*. 2018, 2020, 2024; Novelli *et al*. 2019; Petit *et al*. 2021; Walker *et al*. 2022). From the published literature on *Xylella fastidiosa*, not only on olive trees (Sabella *et al*. 2019; Petit *et al*. 2021; Walker *et al*. 2022) but also on other host species (Chatelet *et al*. 2011; Deyett *et al*. 2019), cultivars resistant/tolerant to the bacterium are characterized by smaller vessels compared with the larger vessels of the susceptible plants. According to the available evidence, this resistance has two possible explanations: first, a smaller vessel can be more quickly compartmentalized by active plant responses (production of tylosis, gums and gels) confining the pathogen to a few vessels and, so, slowing its systemic spread within the host plant (Pouzoulet *et al*. 2019, 2020); the second explanation is that smaller vessels are less susceptible to the embolism phenomenon that can occur in the presence of vascular pathogens, exacerbating symptoms and disease progression (Sabella *et al*. 2019; Petit *et al*. 2021). Anyway, olive trees also induce the formation of smaller xylem vessels under long‐term drought stress (Rossi *et al*. 2013), a condition to which the *Xfp*‐infected plants are exposed. In accordance with this observation, a reduction in vessel density was found in vegetative recovering Cellina di Nardò sampled in 2023–2024 compared with symptomatic plants from 2016 to 2018 and to the healthy plants sampled in the years 2014 and 2015, and in 2025 from the *Xfp*‐free area of Mirto – Crosia (Cosenza, Italy). As a consequence of the vessel density reduction, the vulnerability index significantly increased supporting a greater susceptibility to cavitation in Cellina di Nardò plants in vegetative recovery. However, it is important to consider that plant species displayed great variability in the xylem vessel density variation in response to biotic and abiotic stress, as vessel density integrates many xylem traits contributing to adjust the trade‐off between the water supply system and wood anatomical features (Jacobsen *et al*. 2007; Rodríguez‐Ramírez *et al*. 2022). Among the anatomical features that deserve consideration, there is the vessel re‐arrangement, but the role of the spatial distribution of xylem vessels in the hydraulic system of plants has received limited attention in the past (Scholz *et al*. 2013). The V_G_, V_S_ and V_M_ demonstrate that Cellina di Nardò 1‐year‐old branches harvested in 2023–2024 had considerably more solitary vessels in their xylem than 1‐year‐old branches of symptomatic plants sampled in 2016–2018 and of the constitutive healthy plants in the years 2014–2015 and in 2025 from the disease‐free area of Mirto – Crosia (Cosenza, Italy) (Tables 1, 2, 3; Fig. 7, Figs. S2– S4). This evidence seems to have a substantial explanation; in fact, intervessel connections are primarily dependent on the closeness of adjacent vessels (Brodersen *et al*. 2013). It means that grouped vessels have more tangential connections, boosting xylem network interactions; this feature, while playing a crucial function in water and nutrient transport in plants, nevertheless, can promote the spread of vascular infections (Brodersen *et al*. 2013). In this perspective, it is clear that a plant with more solitary vessels can limit the spread of the pathogen confining it in few vessels and exploiting the functionality of the remaining healthy vessels to regenerate the canopy. This indication has been supported by the observation of the *Xfp* pattern distribution in xylem vessels of the analysed 1‐year‐old branches using fluorescence *in situ* hybridization, because the acquired images showed a reduction of bacterial vessel occlusions in solitary vessels compared with grouped vessels (Fig. 8). In 2013, Brodersen et al. studied the importance of communication between xylem vessels in *Vitis* species susceptible to infection, proposing that the anatomical characteristics of the vessels may contribute to disease and embolism resistance. However, a recent study authored by Fanton *et al*. (2024) indicated that the impact of xylem network features in the distribution of *Xf* across the *Vitis* xylem is modest.

The analysis of plant response to long‐term stress is usually focused on abiotic stress, and the dendro‐anatomical study is widely recognized for examining the correlation of distinct xylem anatomical traits with temperature and precipitation. Because the initial outbreak of *Xfp* in Italy dates from before 2013 (when the first presence of *Xfp* was detected in olive trees of the Apulia region), the survived olive trees have had more than 10 years to adapt, and somehow, try to resist *Xfp*. The increase in solitary narrower vessels provides the host plants with the ability to contain the vascular pathogen, becoming a determinant of disease resistance. It is yet to be determined the causes of this adaptive response, regarding both symptoms and xylem structure, and if the phenomenon is transient, potentially resulting from years of significant pathogen stress, or whether it can persist despite future variations in bacterial levels/annual infectious occurrences that susceptible Cellina di Nardò plants will experience.

As is almost always the case, the data presented in this study do not currently lead to definitive conclusions that need to be confirmed or refuted by other authors who will have the opportunity to read this article.

## AUTHOR CONTRIBUTIONS

ES: Methodology, investigation, data curation, writing—original draft. DG: Investigation, software. EN: Investigation. AF: Investigation. LDB: Supervision, funding acquisition, writing—review and editing. MV: resources. AL: Supervision, writing—review and editing.

## FUNDING INFORMATION

The research received funding from the European Union – Next Generation EU, Mission 4 Component 1, project ‘Identification of Endemic Resources to Contrast *Xylella fastidiosa* ‐ Contra*Xf*’, prot. P2022PW3EB, CUP F53D23011860001; and was partially supported by the Ministero dell'agricoltura, della sovranità alimentare e delle foreste, through the research projects ‘Nuove prospettive di sviluppo per l'olivicoltura italiana attraverso la valorizzazione della biodiversità e la selezione di materiale genetico d'olivo tollerante/resistente a *Xylella fastidiosa* e azioni mirate a prevenire il possibile impatto sulla viticoltura ‐ NOVIXGEN’ (D.D. 664890 del 29 dicembre 2022, CUP C83C22001280006) and ‘Approcci integrati per il miglioramento genetico, la selezione e l'ottenimento di materiali vegetali resistenti a *Xylella fastidiosa* ‐ RIGENERA’ (decreto direttoriale n. 665024 del 29/12/2022, CUP H93C22000750001).

## CONFLICT OF INTEREST

The authors declare that there is no conflict of interest regarding the publication of this article.

## Supporting information


**Fig. S1.** Satellite images by Google Earth for the sampling sites (A–G) in the years 2016, 2017, 2018 and 2023, 2024.

**Fig. S2.** Representative images of the vessel re‐arrangement found in 1 year‐old branch. Columns (A) symptomatic Cellina di Nardò sampled in 2016–2018; columns (B) Cellina di Nardò plant in vegetative recovery sampled in 2023–2024. Red circles highlight grouped vessels; yellow circles mark solitary vessels.

**Fig. S3.** Representative images of the vessel arrangement in 1 year‐old branch of healthy Cellina di Nardò sampled in 2015 from areas with soil and climate characteristics similar to the analysed field (A–G), before the *Xylella fastidiosa* epidemic spread in the Salento area.

**Fig. S4.** Representative images of the vessel arrangement in 1 year‐old branch of healthy Cellina di Nardò sampled in 2025 from the research centre in Mirto – Crosia (Cosenza, Italy) where the CREA‐OFA (Council for Agricultural Research and Economics – Research Centre for Olive, Fruit and Citrus Crops), in agreement with the Regional Department for the Agricultural Development of the Calabria Region (ARSAC), manages the largest collection of olive trees characterized by 405 Italian certified varieties. The area has soil and climate characteristics similar to the analysed field A–G.

**Table S1.** Indices employed to quantify vessel grouping in olive tree branches.

**Table S2.** Average bacterial concentration detected on 1‐year‐old branches of plants sampled in the field sites of: Martano (sites A and B), Leverano (site C), Melendugno (site D), Alimini (sites E and F) and Otranto (site G) during the two sampling periods (symptomatic: years 2016–2018; vegetative recovery: years 2023–2024). Statistical analysis was performed through ANOVA (*P*‐value < 0.05) followed by Tukey‐HSD *post hoc* test. Letter ‘a’ in the table means that the average values in the columns and in the rows do not differ significantly.

## Data Availability

All data generated during the study are included in the manuscript and the Figs. S1– S4 and Tables S1 and S2.
