# Supplementary material for: Resisting Xylella fastidiosa: xylem anatomical changes in the susceptible olive cultivar Cellina di Nardò after long‐term infection
Source: Plant Biol (Stuttg). 2026 Mar 25;28(5):1628–40. doi: 10.1111/plb.70210 (PMC13358715; doi:10.1111/plb.70210)

**Supplemental material**


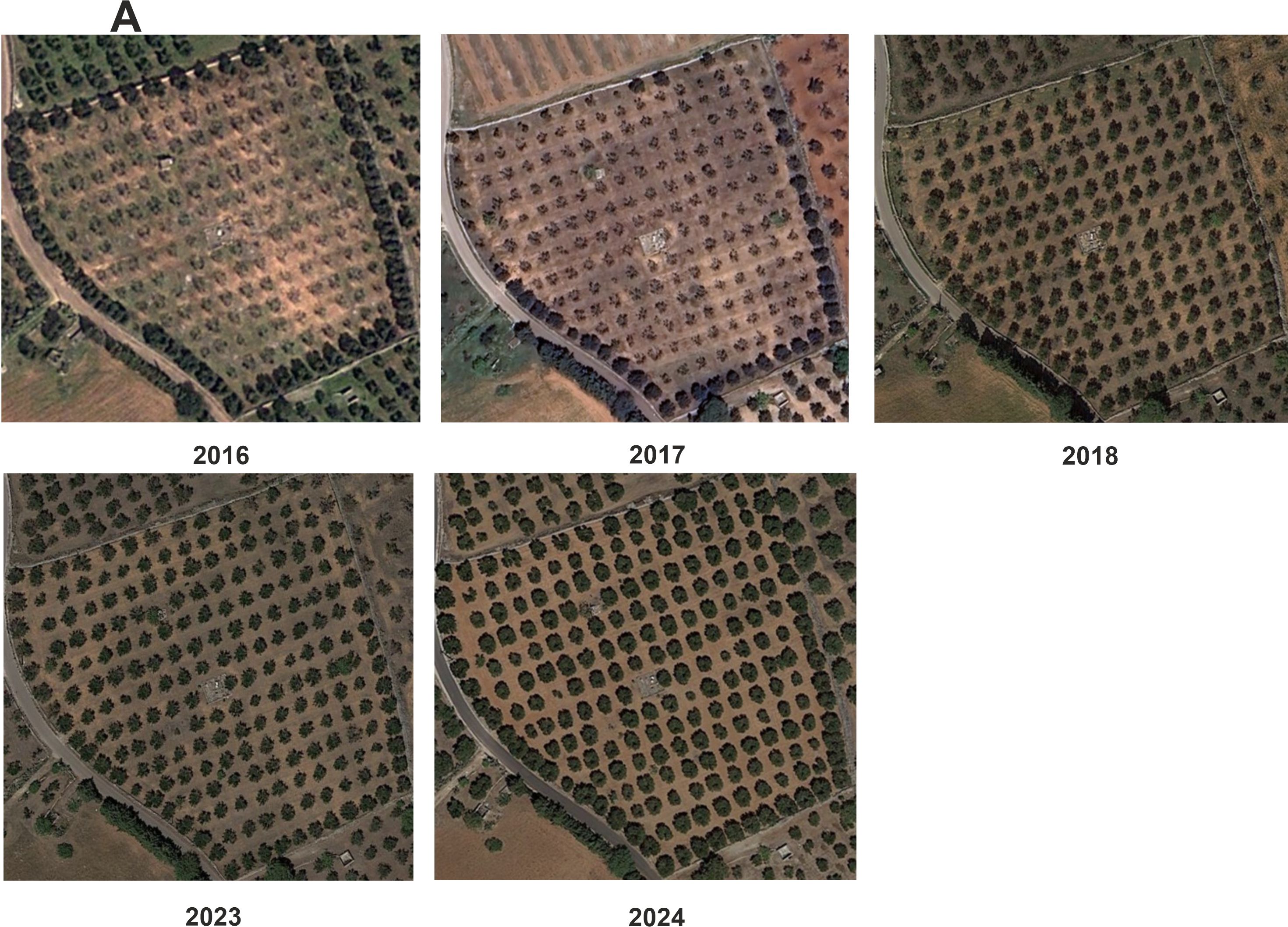
**Figures S1.** Satellite images by Google Earth for the sampling sites A-G in the years 2016, 2017, 2018 and 2023, 2024.


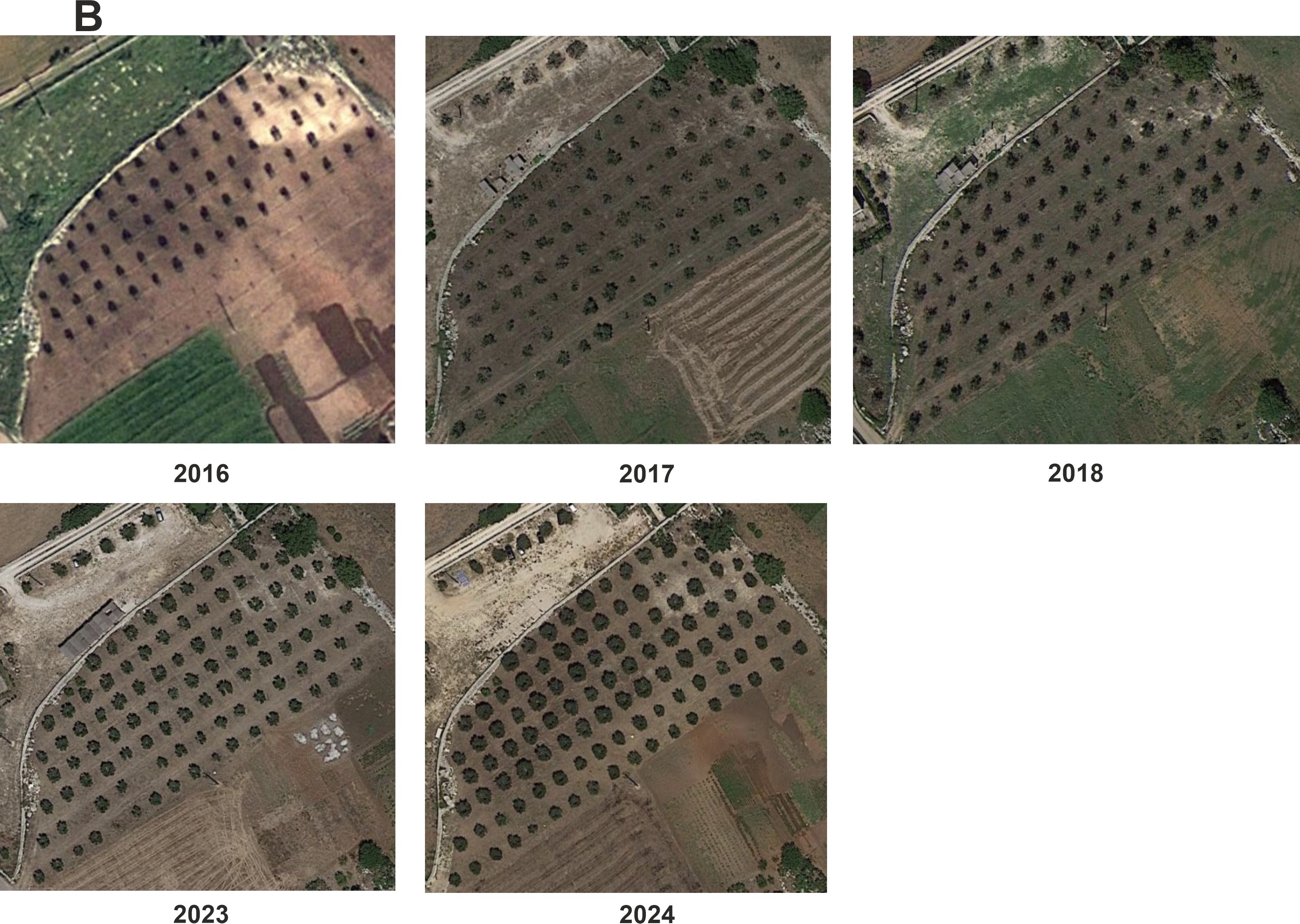


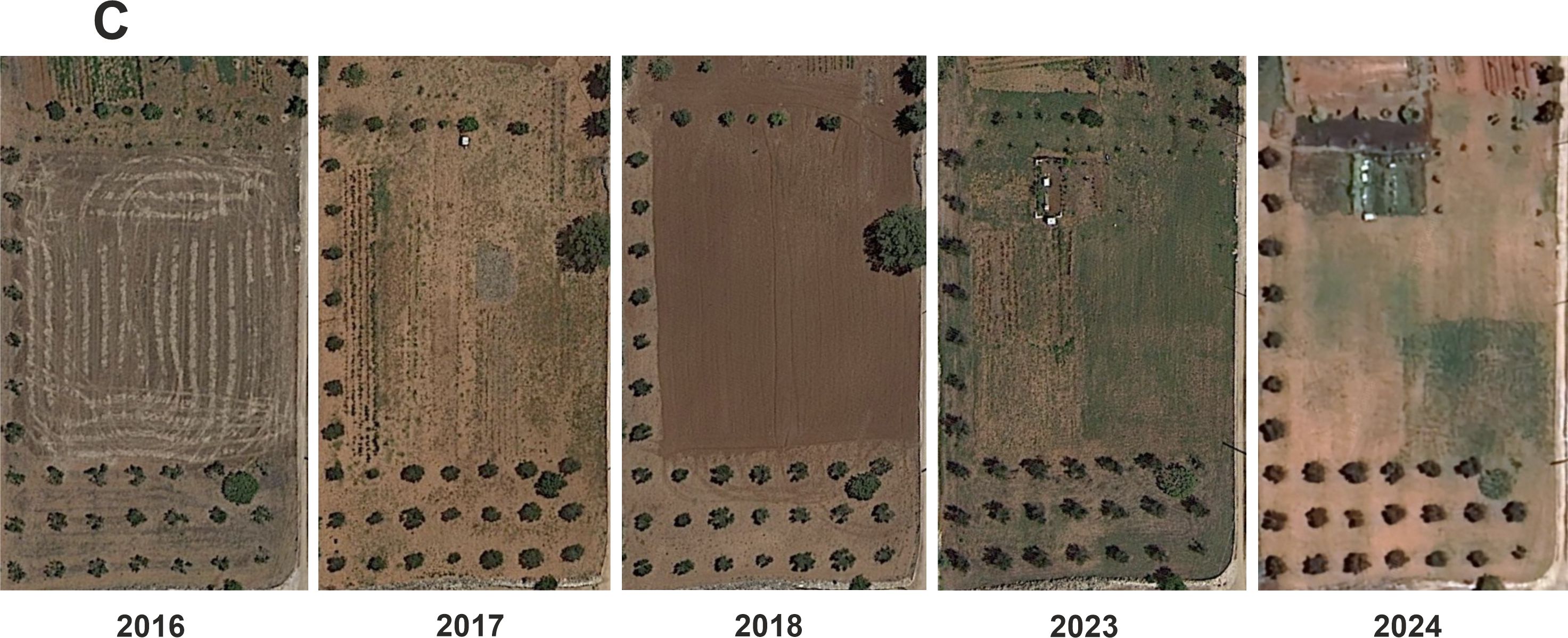


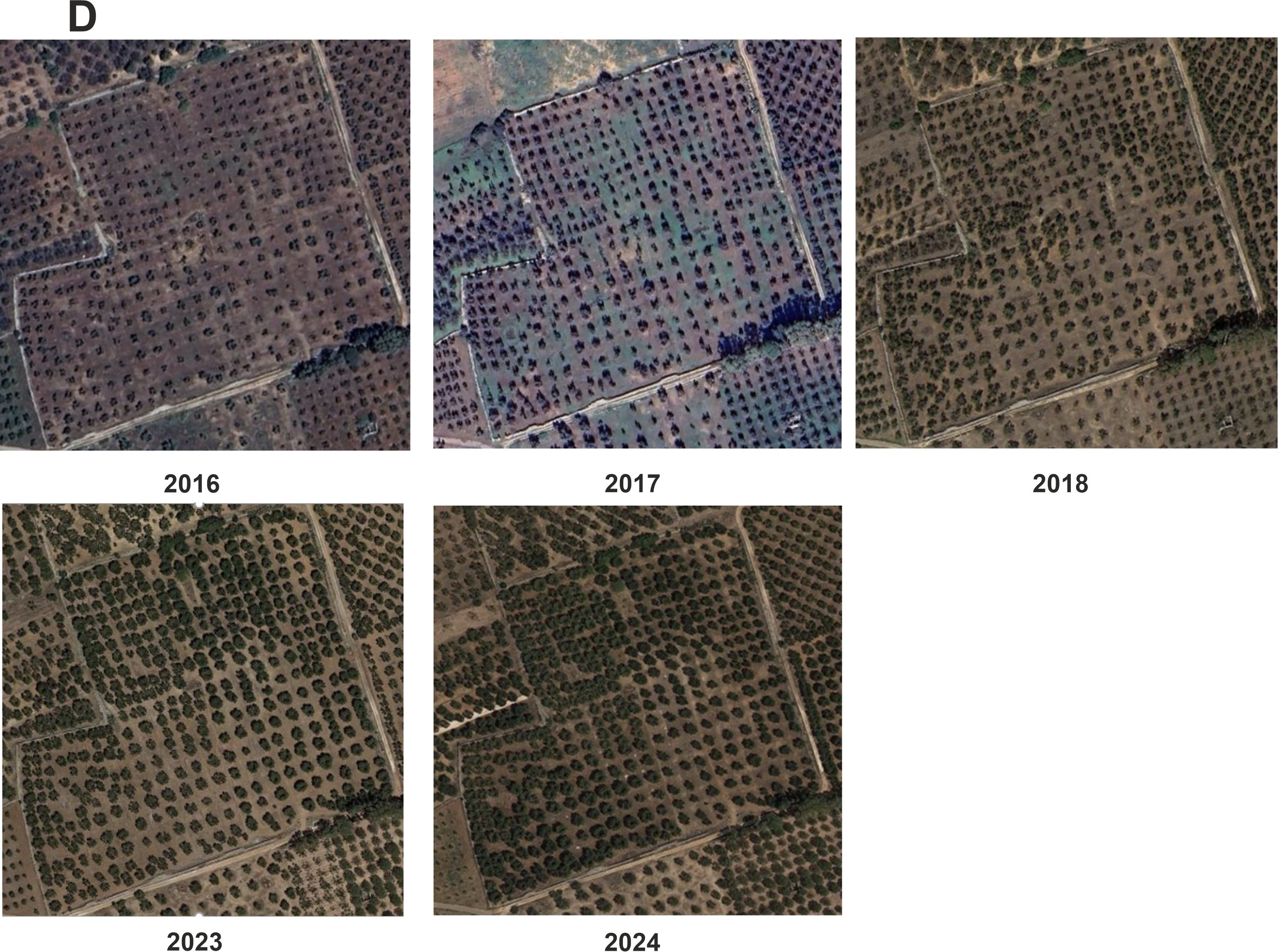


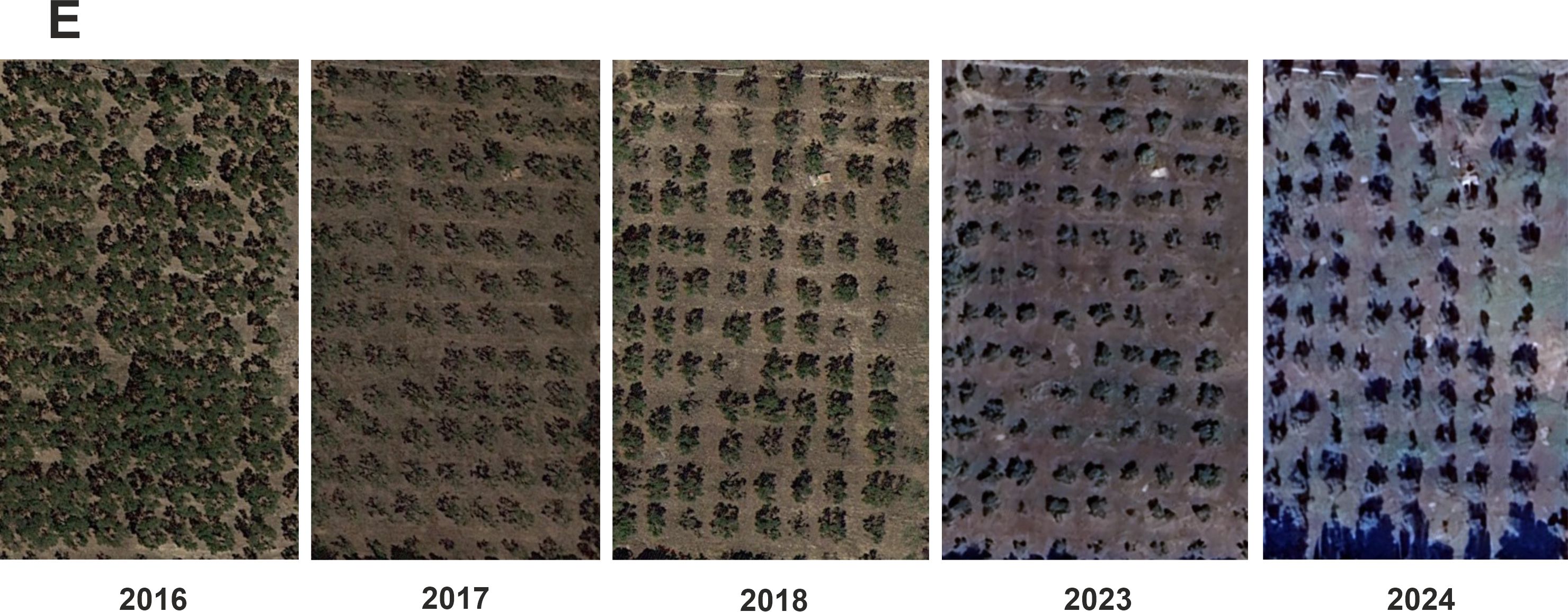


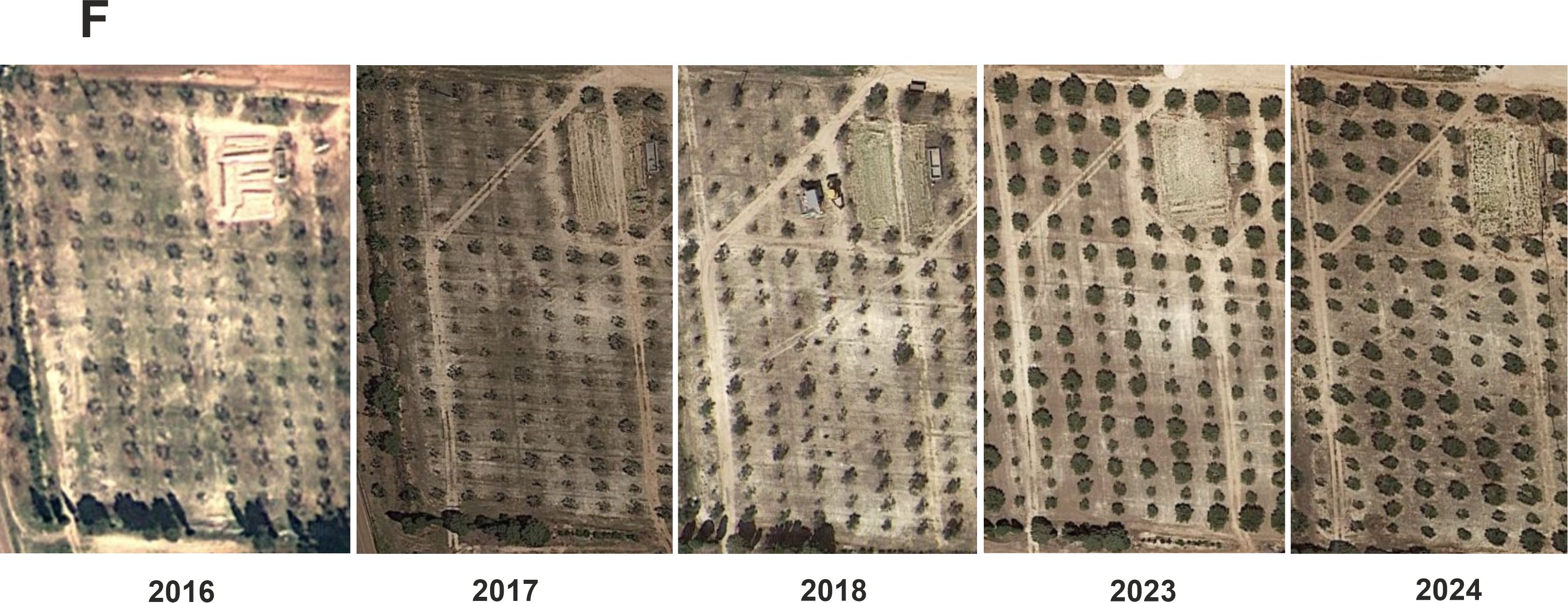


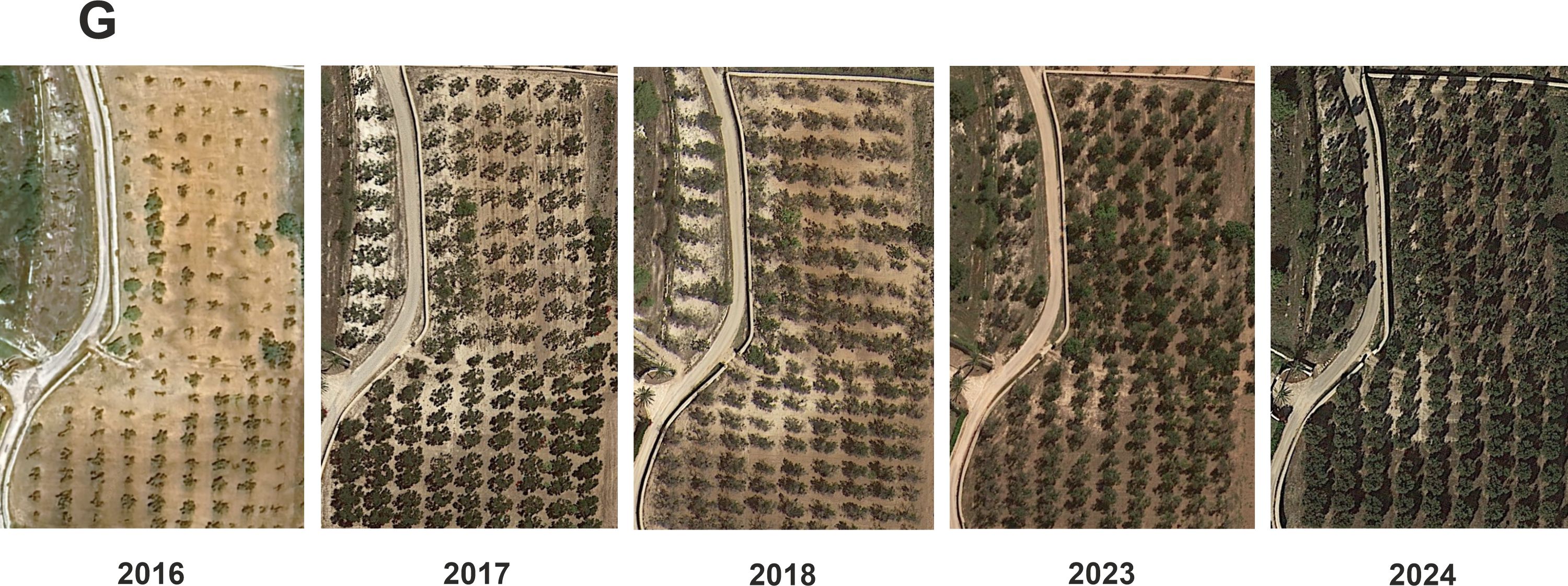

Supplement: Supplementary file 1 — Fig. S1. Satellite images by Google Earth for the sampling sites (A–G) in the years 2016, 2017, 2018 and 2023, 2024. [file PLB-28-1628-s004.docx]
