# Supplementary material for: Resisting Xylella fastidiosa: xylem anatomical changes in the susceptible olive cultivar Cellina di Nardò after long‐term infection
Source: Plant Biol (Stuttg). 2026 Mar 25;28(5):1628–40. doi: 10.1111/plb.70210 (PMC13358715; doi:10.1111/plb.70210)

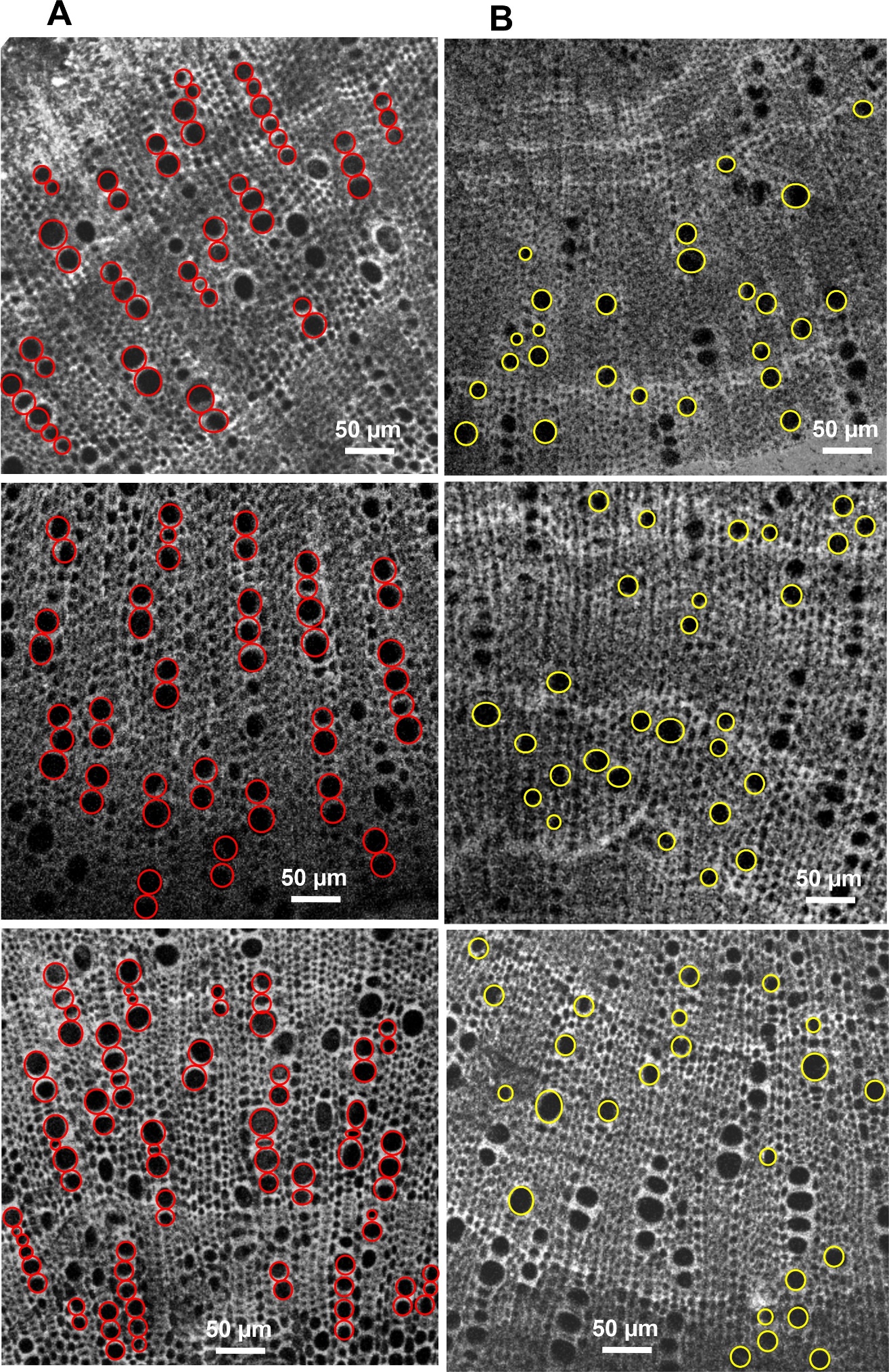
**Figures S2.** Representative images of the vessel re-arrangement found in one year-old branch. Columns A) symptomatic Cellina di Nardò sampled in 2016-2018; columns B) Cellina di Nardò plant in vegetative recovery sampled in 2023-2024. Red circles highlight grouped vessels; yellow circles mark solitary vessels.


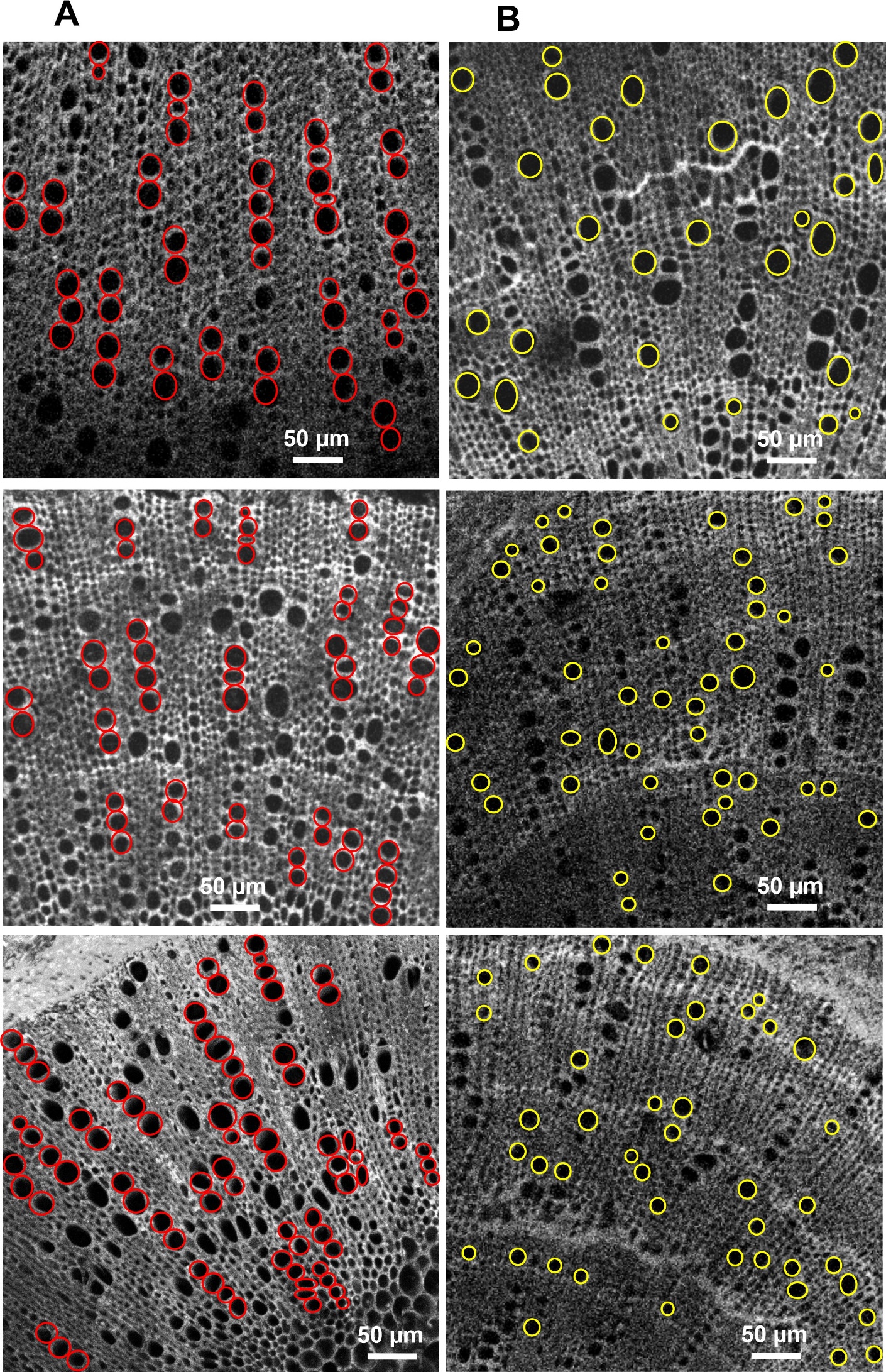

Supplement: Supplementary file 2 — Fig. S2. Representative images of the vessel re‐arrangement found in 1 year‐old branch. Columns (A) symptomatic Cellina di Nardò sampled in 2016–2018; columns (B) Cellina di Nardò plant in vegetative recovery sampled in 2023–2024. Red circles highlight grouped vessels; yellow circles mark solitary vessels. [file PLB-28-1628-s001.docx]
