# Supplementary material for: Resisting Xylella fastidiosa: xylem anatomical changes in the susceptible olive cultivar Cellina di Nardò after long‐term infection
Source: Plant Biol (Stuttg). 2026 Mar 25;28(5):1628–40. doi: 10.1111/plb.70210 (PMC13358715; doi:10.1111/plb.70210)

**Figures S3.** Representative images of the vessel arrangement in one year-old branch of healthy Cellina di Nardò sampled in 2015 from areas with soil and climate characteristics similar to the analysed field A-G, before the *Xylella fastidiosa* epidemic spread in the Salento area.


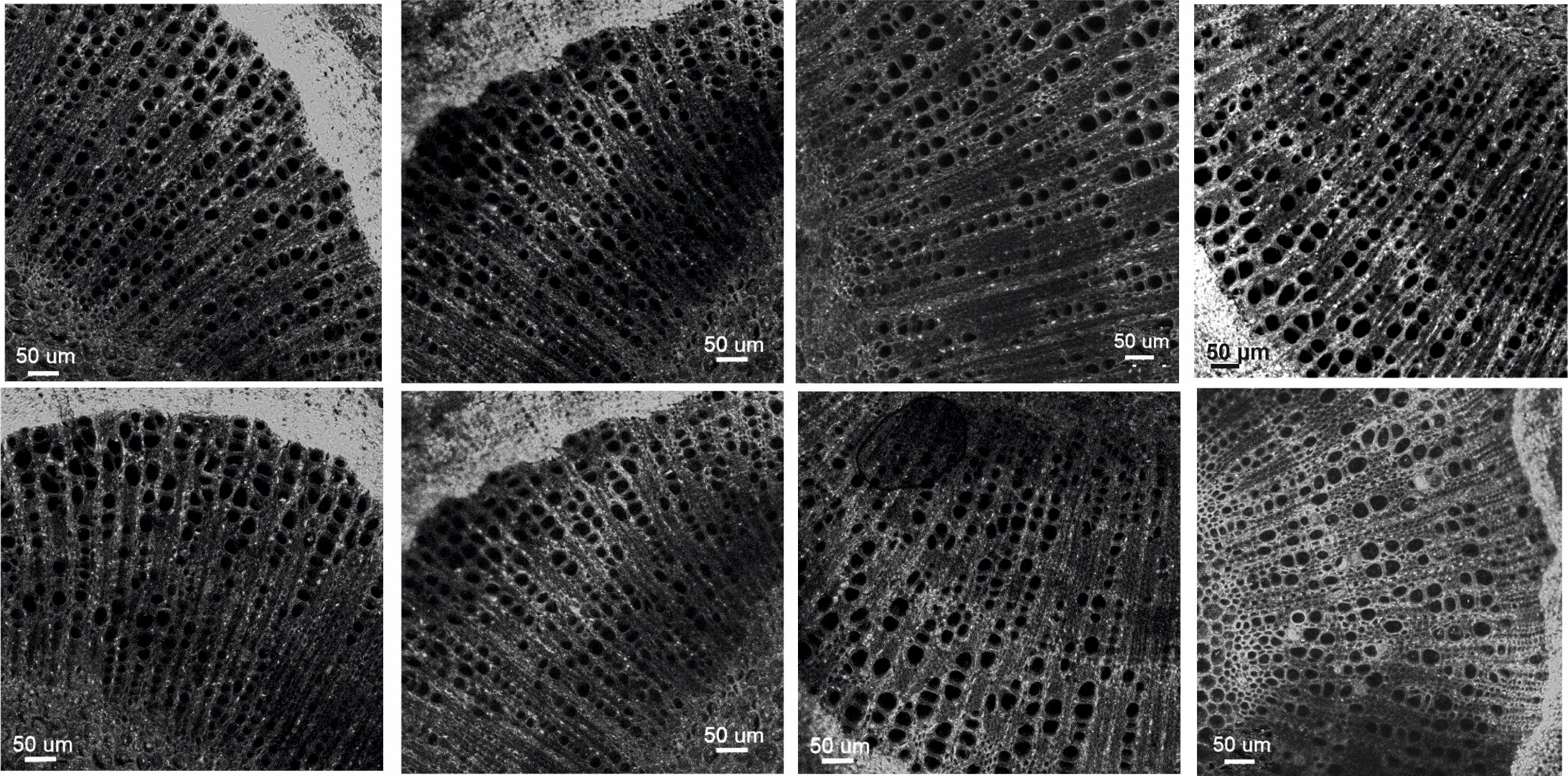

Supplement: Supplementary file 3 — Fig. S3. Representative images of the vessel arrangement in 1 year‐old branch of healthy Cellina di Nardò sampled in 2015 from areas with soil and climate characteristics similar to the analysed field (A–G), before the Xylella fastidiosa epidemic spread in the Salento area. [file PLB-28-1628-s003.docx]
