# Supplementary material for: Resisting Xylella fastidiosa: xylem anatomical changes in the susceptible olive cultivar Cellina di Nardò after long‐term infection
Source: Plant Biol (Stuttg). 2026 Mar 25;28(5):1628–40. doi: 10.1111/plb.70210 (PMC13358715; doi:10.1111/plb.70210)

**Figures S4.** Representative images of the vessel arrangement in one year-old branch of healthy Cellina di Nardò sampled in 2025 from the research centre in Mirto – Crosia (Cosenza, Italy) where the CREA-OFA (Council for Agricultural Research and Economics - Research Centre for Olive, Fruit and Citrus Crops), in agreement with the Regional Department for the Agricultural Development of the Calabria Region (ARSAC), manages the largest collection of olive trees characterized by 405 Italian certified varieties. The area has soil and climate characteristics similar to the analysed field A-G.


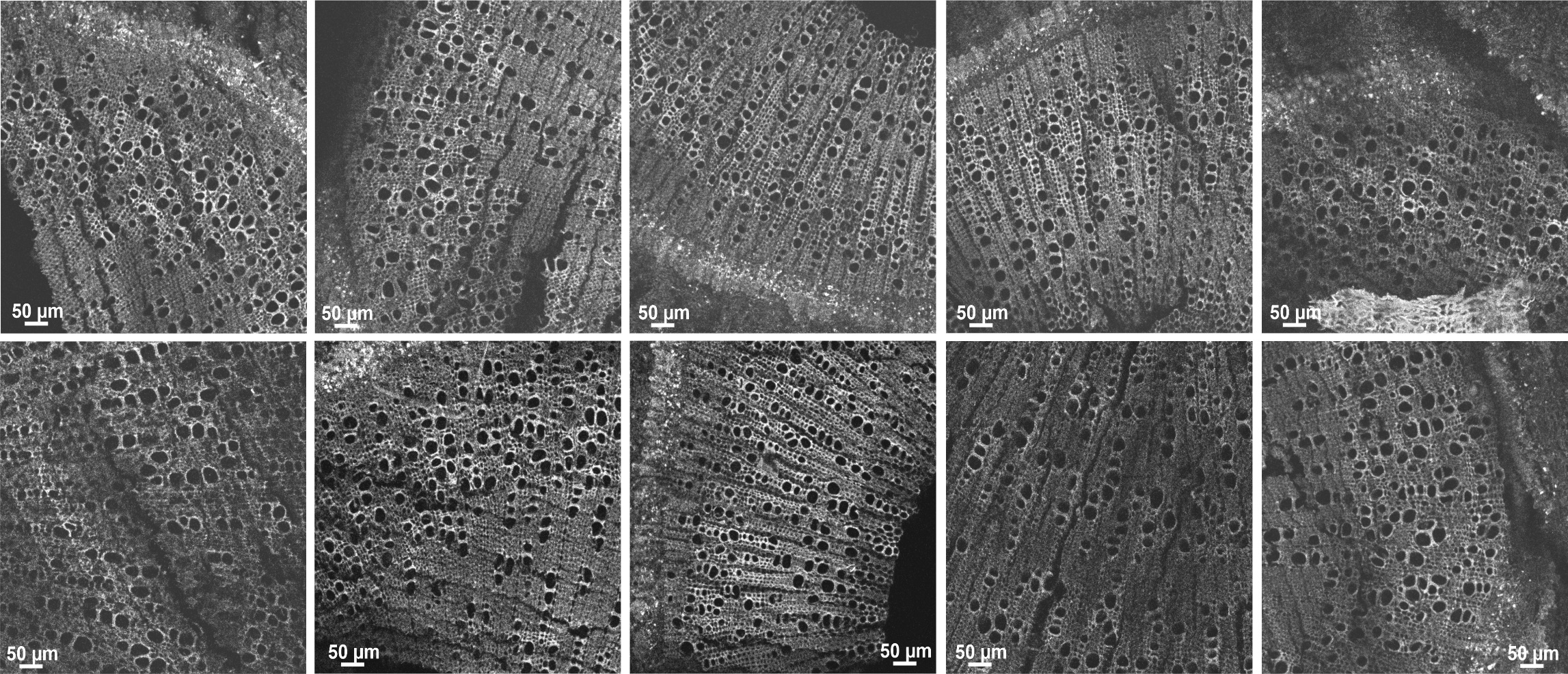

Supplement: Supplementary file 4 — Fig. S4. Representative images of the vessel arrangement in 1 year‐old branch of healthy Cellina di Nardò sampled in 2025 from the research centre in Mirto – Crosia (Cosenza, Italy) where the CREA‐OFA (Council for Agricultural Research and Economics – Research Centre for Olive, Fruit and Citrus Crops), in agreement with the Regional Department for the Agricultural Development of the Calabria Region (ARSAC), manages the largest collection of olive trees characterized by 405 Italian certified varieties. The area has soil and climate characteristics similar to the analysed field A–G. [file PLB-28-1628-s006.docx]
