# Supplementary material for: Resisting Xylella fastidiosa: xylem anatomical changes in the susceptible olive cultivar Cellina di Nardò after long‐term infection
Source: Plant Biol (Stuttg). 2026 Mar 25;28(5):1628–40. doi: 10.1111/plb.70210 (PMC13358715; doi:10.1111/plb.70210)
Supplement: Supplementary file 5 — Table S1. Indices employed to quantify vessel grouping in olive tree branches. [file PLB-28-1628-s002.docx]

**Table S1.** Indices employed to quantify vessel grouping in olive tree branches.

| **Index** |  | **Description** | **References** |
| --- | --- | --- | --- |
| *Vulnerability index* (VI) | *mean vessel* **∅**  *N vessels mm^-2^* | Ratio between mean vessel diameter (μm) and number of vessels per mm^2^ | Carlquist, 1977 |
| *Vessel grouping index* (V_G_) | *N vessels*  *N groupings* | Mean number of vessels  per group | Carlquist, 2001 |
| *Vessel solitary index* (V_S_) | *N solitary vessels*  *N vessels* | Ratio of solitary vessels  to all vessels | Scholz et al., 2013; von Arx et al., 2013 |
| *Vessel multiple index* (V_M_) | *N multiple vessels*  *N multiple groupings* | Mean group size of  non solitary vessels | Scholz et al., 2013; von Arx et al., 2013 |

Reference

von Arx G., Kueffer C., Fonti P. (2013) Quantifying plasticity in vessel grouping–added value from the image analysis tool ROXAS. IAWA Journal, 34 , 433 – 445 . https://doi.org/10.1163/22941932-00000035
