# Supplementary material for: Resisting Xylella fastidiosa: xylem anatomical changes in the susceptible olive cultivar Cellina di Nardò after long‐term infection
Source: Plant Biol (Stuttg). 2026 Mar 25;28(5):1628–40. doi: 10.1111/plb.70210 (PMC13358715; doi:10.1111/plb.70210)
Supplement: Supplementary file 6 — Table S2. Average bacterial concentration detected on 1‐year‐old branches of plants sampled in the field sites of: Martano (sites A and B), Leverano (site C), Melendugno (site D), Alimini (sites E and F) and Otranto (site G) during the two sampling periods (symptomatic: years 2016–2018; vegetative recovery: years 2023–2024). Statistical analysis was performed through ANOVA (P‐value < 0.05) followed by Tukey‐HSD post hoc test. Letter ‘a’ in the table means that the average values in the columns and in the rows do not differ significantly. [file PLB-28-1628-s005.docx]

**Table S2.** Average bacterial concentration detected on one-year-old branches of plants sampled in the field sites of: Martano (sites A and B), Leverano (site C), Melendugno (site D), Alimini (sites E and F) and Otranto (site G) during the two sampling periods (symptomatic: years 2016-2018; vegetative recovery: years 2023-2024). Statistical analysis was performed through ANOVA (p-value < 0.05) followed by Tukey-HSD post hoc test. Letter “a” in the table means that the average values in the columns and in the rows do not differ significantly.

| **Field site** |  | |  |  | ***Xfp* concentration (cfu/mL)** | | |
| --- | --- | --- | --- | --- | --- | --- | --- |
|  | **2016** | **2017** | | **2018** | | **2023** | **2024** |
| **A** | 1.23 × 10^6^ a | 5.15 × 10^4^ a | | 1.30 × 10^5^ a | | 9.38 × 10^4^ a | 2.44 × 10^5^ a |
| **B** | 9.64 × 10^4^ a | 5.29 × 10^5^ a | | 2.02 × 10^6^ a | | 3.54 × 10^5^ a | 2.56 × 10^6^ a |
| **C** | 3.13 × 10^6^ a | 1.19 × 10^5^ a | | 7.23 × 10^6^ a | | 1.39 × 10^5^ a | 3.28 × 10^6^ a |
| **D** | 8.58 × 10^4^ a | 2.73× 10^4^ a | | 8.57 × 10^6^ a | | 2.10 × 10^5^ a | 1.81 × 10^6^ a |
| **E** | 6.34 × 10^5^ a | 4.60 × 10^5^ a | | 3.77 × 10^7^ a | | 5.18 × 10^5^ a | 3.11 × 10^5^ a |
| **F** | 3.87 × 10^5^ a | 6.83 × 10^5^ a | | 1.50 × 10^5^ a | | 8.16 × 10^4^ a | 4.07 × 10^4^ a |
| **G** | 6.59 × 10^5^ a | 1.01 × 10^7^ a | | 7.37 × 10^7^ a | | 2.74 × 10^4^ a | 9.18 × 10^6^ a |
